# Biomarkers and potential therapeutic targets driving progression of non-alcoholic steatohepatitis to hepatocellular carcinoma predicted through transcriptomic analysis

**DOI:** 10.3389/fimmu.2024.1502263

**Published:** 2024-12-04

**Authors:** Hui Fan, Rong Wang, Bin Wen, Jing Xiong

**Affiliations:** ^1^ Department of Pharmacology, School of Pharmacy, China Pharmaceutical University, Nanjing, China; ^2^ State Key Laboratory of Natural Medicines, School of Traditional Chinese Pharmacy, China Pharmaceutical University, Nanjing, China

**Keywords:** non-alcoholic steatohepatitis, triggering receptor expressed on myeloid cells 2, Annexin A2, growth/differentiation factor-15, tetratricopeptide repeat domain 39A, hepatocellular carcinoma, bioinformatic analysis

## Abstract

**Background:**

Non-alcoholic steatohepatitis (NASH) is the most prevalent chronic liver condition globally, with potential progression to cirrhosis, and even hepatocellular carcinoma (HCC). The increasing prevalence of NASH underscores the urgent need for advanced diagnostic and therapeutic strategies. Despite its widespread impact, effective treatments to prevent the progression of NASH remain elusive, highlighting the critical importance of innovative molecular techniques in both the diagnosis and management of this disease.

**Methods:**

Six microarray datasets available in GEO were used to perform Robust Rank Aggregation (RRA) to identify differentially expressed genes (DEGs).We identified 62 robust upregulated genes and 24 robust downregulated genes. These genes were undergone Gene Ontology enrichment analysis and further examination for expression correlation with NAS score. Molecular subtypes were generated using “ConsensusClusterPlus” on identified genes, which were further assessed for tumor stage relevance, expression differences in adjacent and tumor tissues, and impact on survival in TCGA liver cancer patients. Single-cell analysis was then used to explore the genes across different cell types and subgroups as well as cell-type interactions. The clinical utility of predicted core genes was highlighted through decision curve analysis, with emphasis on HCC prognosis. The GDSC database was used to evaluate the relationship between the predicted core genes and drug sensitivity, while the TIDE database was used to evaluate their relationship with immunotherapy.

**Results:**

Four core genes, *TREM2*, *GDF15*, *TTC39A*, and *ANXA2*, were identified as key to influencing HCC prognosis and therapy responsiveness, especially immune treatment efficacy in NASH-associated HCC.

**Conclusion:**

The core genes may act as critical biomarkers driving the progression of NASH to HCC. They are potential novel targets for the diagnosis and treatment of NASH progression, offering innovative perspectives for its clinical management.

## Introduction

Non-alcoholic fatty liver disease (NAFLD) is a significant global health challenge due to its 20–25% global prevalence and lack of approved targeted therapies (1, 2). NAFLD involves excessive triglyceride accumulation in hepatocytes, progressing from simple steatosis (NAFL) to non-alcoholic steatohepatitis (NASH). This progression can irreversibly advance to cirrhosis and eventually hepatocellular carcinoma (HCC), driven by metabolic alterations and toxic metabolite accumulation (3–5). NASH is unique among the various etiologies of HCC as it involves chronic hepatitis, necroinflammation, and complex metabolic dysregulation (6). NASH-related HCC is a significant public health issue with its incidence rising alongside obesity, diabetes, and metabolic syndrome. It accounts for 2% of global HCC cases, accentuating the critical need for specific therapeutic interventions, which is currently deficient (7, 8).

In terms of treatment, current strategies primarily rely on managing metabolic deregulation of NASH such as weight reduction, improving insulin resistance, and treating hyperglycemia and hyperlipidemia (9). For NASH-progressed HCC, clinical strategies include liver resection, liver transplantation, and local ablation techniques (10). However, these treatments are highly invasive and risky for patients. Chronic inflammation is a central component in the progression of NAFLD to NASH and eventually to HCC (11). Recent studies suggest addressing systemic inflammation to manage the progression of NASH to HCC. Therapeutic agents for inflammatory pathways, such as inhibitors of cytokines or interventions modulating immune cell activity, are being explored (12). Thus, understanding the molecular signatures that link inflammation with NASH-associated HCC development will guide the design of predictive biomarkers and targeted therapies, which are critical for early detection and treatment of this severe liver disease.

The immune system, both adaptive and innate responses, plays a critical role in the progression of NAFL to NASH and eventually to HCC (13). Inflammatory cytokines, such as tumor necrosis factor-α and interleukin-6, and immune cells contribute to hepatic injury and cell transformation, leading to cancerous changes (14, 15). In a mouse model of NASH induced by a choline-deficient high-fat diet, the simultaneous activation of both CD8^+^ T cells and natural killer T cells accelerates hepatic tumor development (16). Concurrently, an increase in hepatic CD8^+^PD1^+^ T cells impairs immune surveillance, thereby initiating liver cancer (17). The chronic inflammation associated with NAFLD also results in the inhibition of cytotoxic CD8^+^ T cells by IgA^+^ cells, interrupting immune surveillance and facilitating the progression of HCC (18). Conversely, CD4^+^ T cells are essential for effective immune monitoring and are recognized for their role in hindering HCC tumor growth (19). However, a targeted reduction of CD4^+^ T cells in MYC oncogene transgenic mice on a methionine-choline-deficient diet results in the development of HCC tumors (20). Recently, immunotherapy, especially checkpoint inhibitors, has shown potential in treating NASH-HCC. Immune checkpoint inhibitors are believed to reestablish tumor immune surveillance by acting on the programmed cell death-1 receptor (PD1) on exhausted CD8^+^ T cells, or the programmed cell death 1 ligand 1 (PD-L1) on tumor cells (18, 21–23). However, the treatment effects remain individually variant due to the highly personalized inflammatory environment of NASH-HCC.

Future drug development is expected to increasingly target liver-specific pathologies, such as unique inflammatory signaling pathways, apoptosis processes, and the gut-liver axis regulation (24). Therefore, a better understanding of the hepatic microenvironment is pivotal for developing therapeutics that modulate the immune response, particularly by identifying key genes as potential biomarkers or drug targets in the progression from NASH to HCC. In this study, the Robust Rank Aggregation (RRA) method was employed to identify genes consistently expressed in the NASH model and to ascertain their correlation with NAS scores in human datasets. Subsequently, these genes were investigated within the context of liver cancer, leading to the identification of key genes correlated with patient survival rates. Besides, we analyzed cell-type interactions within the NASH model and utilized Decision Curve Analysis to predict drug sensitivity targeting these genes based on risk stratification. Finally, we evaluated the potential of these genes as targets for immunotherapy in patients with NASH-associated HCC. In brief, our findings provide new insights and a theoretical framework for targeted therapy in NASH-associated HCC.

## Materials and methods

### Data collection and processing

**Figure 1:**
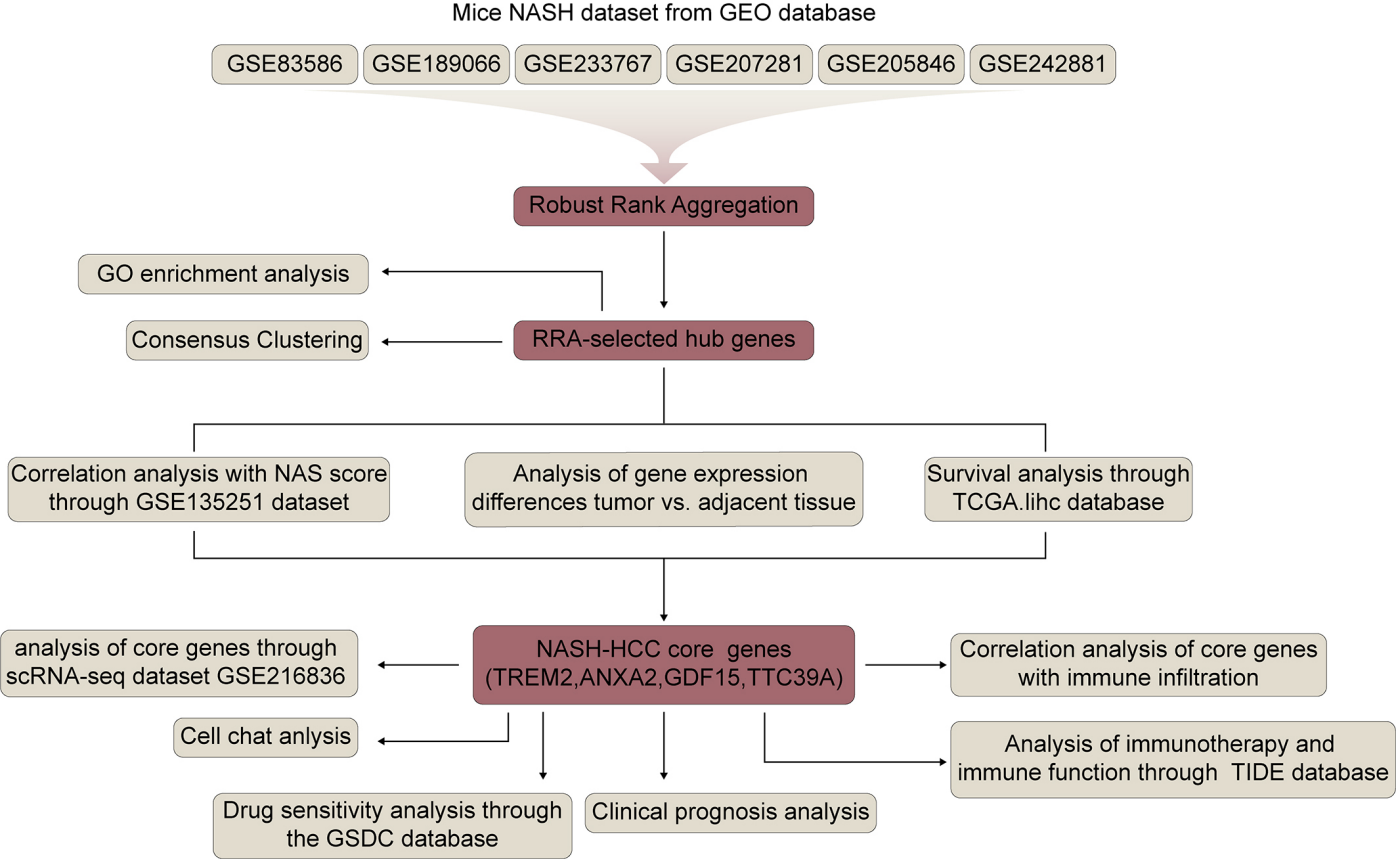
Flowchart of the study.

**Figure 1** shows the flowchart of this study. Six mice NASH datasets were retrieved and downloaded from Gene Expression Omnibus (GEO) database (https://www.ncbi.nlm.nih.gov/geo/). GSE83596 consists of 32 samples: 3 steatosis stage control, 3 NASH stage control, 4 fibrosis stage control, 4 tumor stage control, 3 steatosis, 3 NASH, 4 fibrosis, 4 non-tumor, 4 tumor. GSE189066 consists of 6 samples: 3 control, 3 NASH. GSE233767 consists of 8 samples: 4 control, 4 NASH. GSE207281 contains 3 control, 3 NASH. GSE205846 consists of 8 samples: 4 control, 4 NASH. GSE242881 contains 3 control, 3 NASH. GSE246221 contains 5 control, 14 NASH-associated HCC. Human NASH dataset GSE135251 was also downloaded from GEO database and it contains 10 control, 11 NAFLD patients with NAS score 1, 21 NAFLD patients with NAS score 2, 26 NAFLD patients with NAS score 3, 38 NAFLD patients with NAS score 4, 47 NAFLD patients with NAS score 5, 37 NAFLD patients with NAS score 6, 18 NAFLD patients with NAS score 7, 8 NAFLD patients with NAS score 8. All bulk-seq data were processed using R language and the fold change between control and NASH were calculated. Identification of differentially expressed genes was performed using the R package, “DESeq2”, and a p value <0.05 was used to identify the differentially expressed genes (DEGs).

The analysis of single-cell sequencing data utilized publicly available samples from GEO (GSE216836), including two controls and six NASH samples, and was processed in R. To identify cellular subpopulations in single-cell RNA sequencing (scRNA-seq) data, we used the Seurat package (version 4.3.0.1) for data processing. The workflow involved the following steps:1) Data Conversion and Quality Control: We converted 10x Genomics scRNA-seq data into Seurat objects using the Seurat package. Quality control was performed before specific analyses to ensure data integrity. We retained genes that were expressed in at least three cells and kept cells with at least 200 detected genes. Using the PercentageFeatureSet function, we calculated the percentage of mitochondrial content, ensuring that it was below 25%. 2) Normalization and Variable Gene Selection: We used the NormalizeData function to normalize gene expression, reducing technical noise and enhancing downstream analysis accuracy. We then identified the top 2000 highly variable genes using the FindVariableFeatures function. 3) Principal Component Analysis (PCA): We performed PCA to reduce data dimensionality and capture dominant data signals for downstream analysis. 4) Batch Effect Correction and Data Integration: To correct batch effects, we used the RunHarmony function from the Harmony R package, integrating data based on PCA results. 5) Further Dimensionality Reduction and Clustering: Using the Harmony-corrected dimensionality reduction results, we performed cluster analysis and visualized the data. UMAP was applied to further reduce dimensionality and visualize the integrated data, revealing distinct cell clusters. 6) Cell Type Annotation: Finally, cell clusters were annotated using the CellMarker database (http://xteam.xbio.top/CellMarker/) to assign cell types based on known marker genes.

### Robust rank aggregation analysis

Robust Rank Aggregation (RRA) was used to further identify robust DEGs from different datasets in an unbiased manner using a comprehensive ranking list algorithm, and a p value of <0.01 and log| FC| > 1 were considered to indicate significance (25).

### Biological function and pathway enrichment analyses

Gene Set Enrichment Analysis (GSEA) and Gene Ontology (GO) pathway enrichment analysis were conducted using the “ClusterProfiler” R package (26).

### Consensus clustering of RRA-selected hub genes

“ConsensusClusterPlus” R package was applied to calculate how frequently HCC samples were grouped by RRA-selected hub genes. We used the proportion of ambiguously clustered pairs (PAC) to accurately estimate the optimal cluster number (K). Two clusters were identified, and further survival analysis was conducted by the Kaplan-Meier curve with the log-rank test. Principal component analysis (PCA) was performed by the “ggplot2” R package.

### Gene mutation analysis

Gene mutation was conducted through “maftools” package, based on the somatic mutation data from TCGA-LIHC. And then we calculated tumor mutation burden (TMB) of each patient and compared TMB between the high- and low-risk groups. Survival analysis was also performed according to TMB score.

### Hydrodynamic transfection

Hydrodynamic tail vein injection was performed as described in the literature (27). Plasmids were mixed in 2 mL of Normal Saline(NS) and injected into mice via the tail vein within 7 seconds. 12 mice were acclimated and randomly divided into a control group (n=6) and a NASH group (n=6). The model group received a rapid tail vein injection of a 2 mL solution containing 15 μg of ΔN90-beta-catenin, 15 μg of myr-AKT, and 5 μg of pCMV/SB plasmid mix, while the control group was administered an equivalent volume of saline. Mice were anesthetized and euthanized at 4 days, 4 weeks, and 2 months post-injection.

### Western blot

Liver tissue were collected, and protein was extracted using a RIPA lysis buffer, and protein concentrations were measured using BCA (Beyotime, China). The proteins were loaded onto 10–12% SDS-PAGE and transferred onto PVDF membranes, which were blocked using 5% non-fat milk at room temperature for 1 h, and incubated with primary antibodies overnight at 4°C. After washing the membrane with TBST three times, the membrane was incubated with the secondary antibody at room temperature for 1 h. Finally, a chemiluminescence reagent imaging system was used to detect the bands by using the Tanon 4800 system.

### RT-qPCR

Liver tissues from wild-type mice and NASH models were thoroughly homogenized, and RNA was extracted using the TRIzol method. Reverse transcription and PCR were performed using Vazyme’s reverse transcription kit (catalog number R323) and PCR kit (catalog number Q341), respectively. The reverse transcription took place on the GeneAmp PCR System 9700 from Applied Biosystems, and PCR amplification was executed on the LightCycler 480 II system from Roche. All primers were sourced from Tsingke Biotechnology. The primer sequences for RT-PCR are as follows: Rplp0 Forward: GAAACTGCTGCCTCACATCCG, Reverse: GCTGGCACAGTGACCTCACACG; *Trem2* Forward: CAGCACCTCCAGGAATCAAGA, Reverse: AGGATCTGAAGTTGGTGCCC; *Gdf15* Forward: CTGGCAATGCCTGAACAACG, Reverse: GGTCGGGACTTGGTTCTGAG; *Anxa2* Forward: GTGCCTACGGGTCAGTCAAA, Reverse: CACATTGCTGCGGTTTGTCA; *Ttc39a* Forward: CAGAAGGGCCACAAGGACTC, Reverse: AATCCTGGTGGGAAGCATGG.

All experiments were performed in triplicate. Melting curve analysis confirmed PCR specificity with single peaks. Ct values were analyzed using the 2^(-ΔΔCt) method, with Rplp0 as the reference, to calculate relative RNA expression levels.

### Drug susceptibility and immunotherapy responsiveness evaluation of risk signature and related genes

In this study, we utilized the R package “oncoPredict” to assess drug sensitivity in LIHC based on gene expression data from patients. This tool enabled us to evaluate the potential effectiveness of various chemotherapeutic agents by calculating drug sensitivities. Additionally, it allowed for the identification of potential biomarkers that could predict the clinical response to specific treatments. We further explored the associations between these predicted drug responses and patient clinical characteristics to enhance the understanding of therapeutic outcomes in HCC.

The Tumor Immune Dysfunction and Exclusion (TIDE) method evaluates the responsiveness of cancer to immunotherapy using pre-treatment tumor expression profiles. It assesses various transcriptomic biomarkers, including TIDE, Dysfunction, Exclusion, MSI.score, TMB, CD274 and CD8 to predict patient responses to treatments. This approach helps in comparing the effectiveness of different biomarkers based on their predictive power regarding treatment outcomes, aiding in the optimization of immunotherapy strategies.

## Results

### Identification of hub genes in NASH through Robust Rank Aggregation analysis

**Figure 2:**
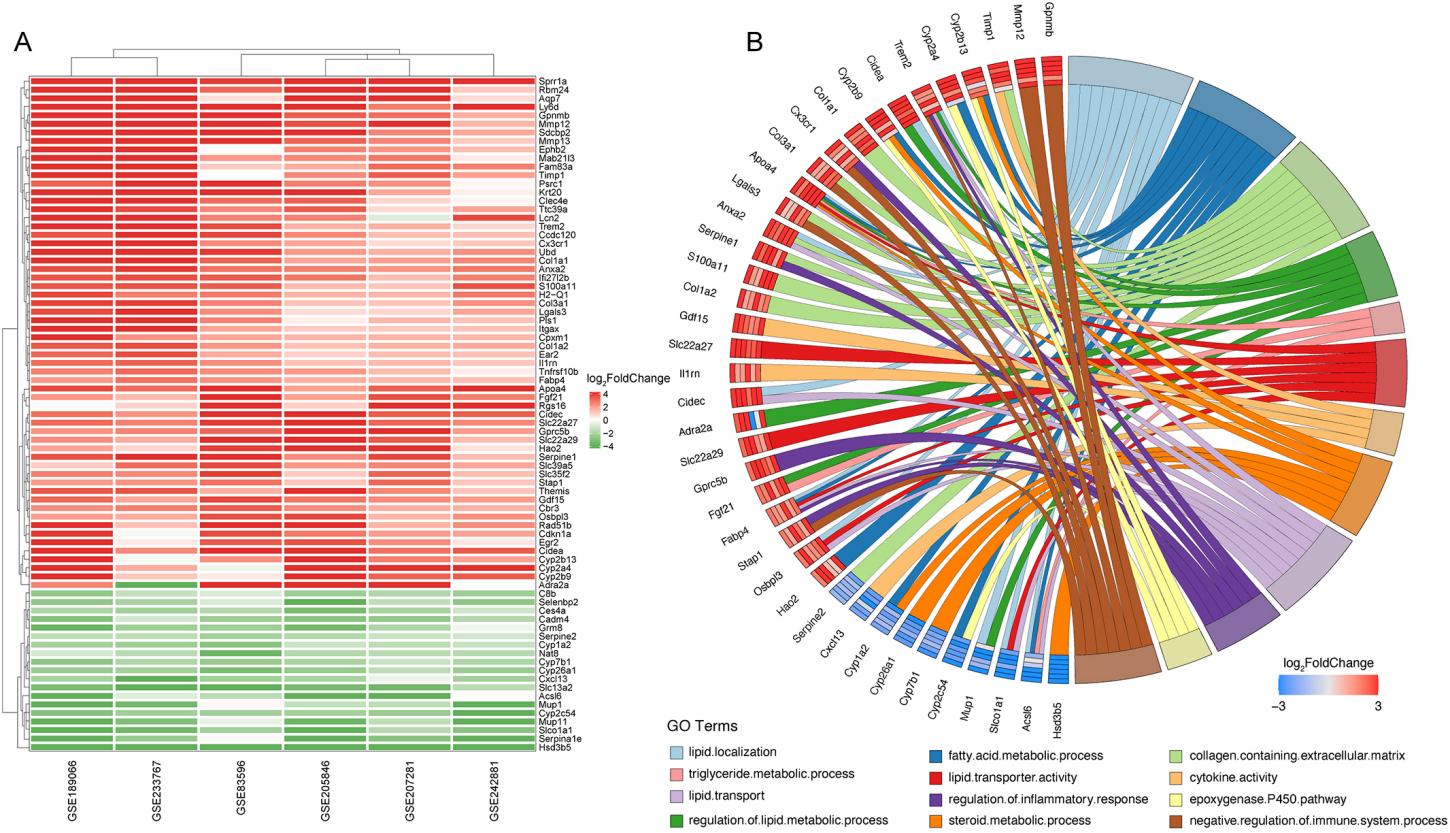
Identification of key differentially expressed genes in murine NASH models using Robust Rank Aggregation method. **(A)** Heatmap of Differentially Expressed Genes (DEGs) in murine NASH models analyzed by Robust Rank Aggregation (RRA). Red: upregulation; Green: downregulation. **(B)** Chord diagram of Gene Ontology (GO) Enrichment Analysis for DEGs in **(A)**.

We conducted a RRA analysis on liver gene expression from six murine models of NASH, identifying 62 upregulated and 24 downregulated genes (**Figure 2A**). The distribution of these genes is illustrated in the volcano plots across the six analyzed GEO datasets (**Supplementary Figure 1**). Gene Ontology (GO) enrichment analysis revealed significant enrichment of these genes in terms related to lipid metabolism and inflammatory processes, including lipid localization, fatty acid metabolic process, collagen-containing extracellular matrix, regulation of lipid metabolic process, triglyceride metabolic process, lipid transporter activity, cytokine activity, steroid metabolic process, lipid transport, regulation of inflammatory response, epoxygenase P450 pathway, and negative regulation of immune system processes (**Figure 2B**). The enriched pathways reveal significant involvement of metabolic processes that are integral to the development and progression of liver diseases leading to HCC. Critical pathways such as lipid localization, lipid transport, and the metabolic processes of fatty acids and triglycerides are highlighted. Dysregulation in these pathways can lead to excessive lipid accumulation, contributing to steatosis, a hallmark of NASH. Furthermore, abnormalities in lipid metabolism are directly linked to oxidative stress and lipotoxicity, which can exacerbate liver damage and promote fibrogenesis. Chronic inflammation is a key driver in the transition from NASH to HCC, and the pathways identified underscore its pivotal role. The activity of cytokines as mediators of inflammation is crucial. Abnormal cytokine profiles can lead to an unresolved inflammatory state, driving the progression of liver diseases. The genes identified through Robust Rank Aggregation (RRA) may influence the progression of disease by impacting these signaling pathways. This suggests a potential mechanism by which these genes could modulate key metabolic and inflammatory processes, thereby affecting the development and progression of liver diseases such as NASH leading to HCC. Understanding these interactions offers insights into how specific gene alterations contribute to disease dynamics and opens avenues for targeted therapeutic interventions.

### Elevated expression of hub genes in liver cancer associated with poor patient survival and altered immune cell profiles

**Figure 3:**
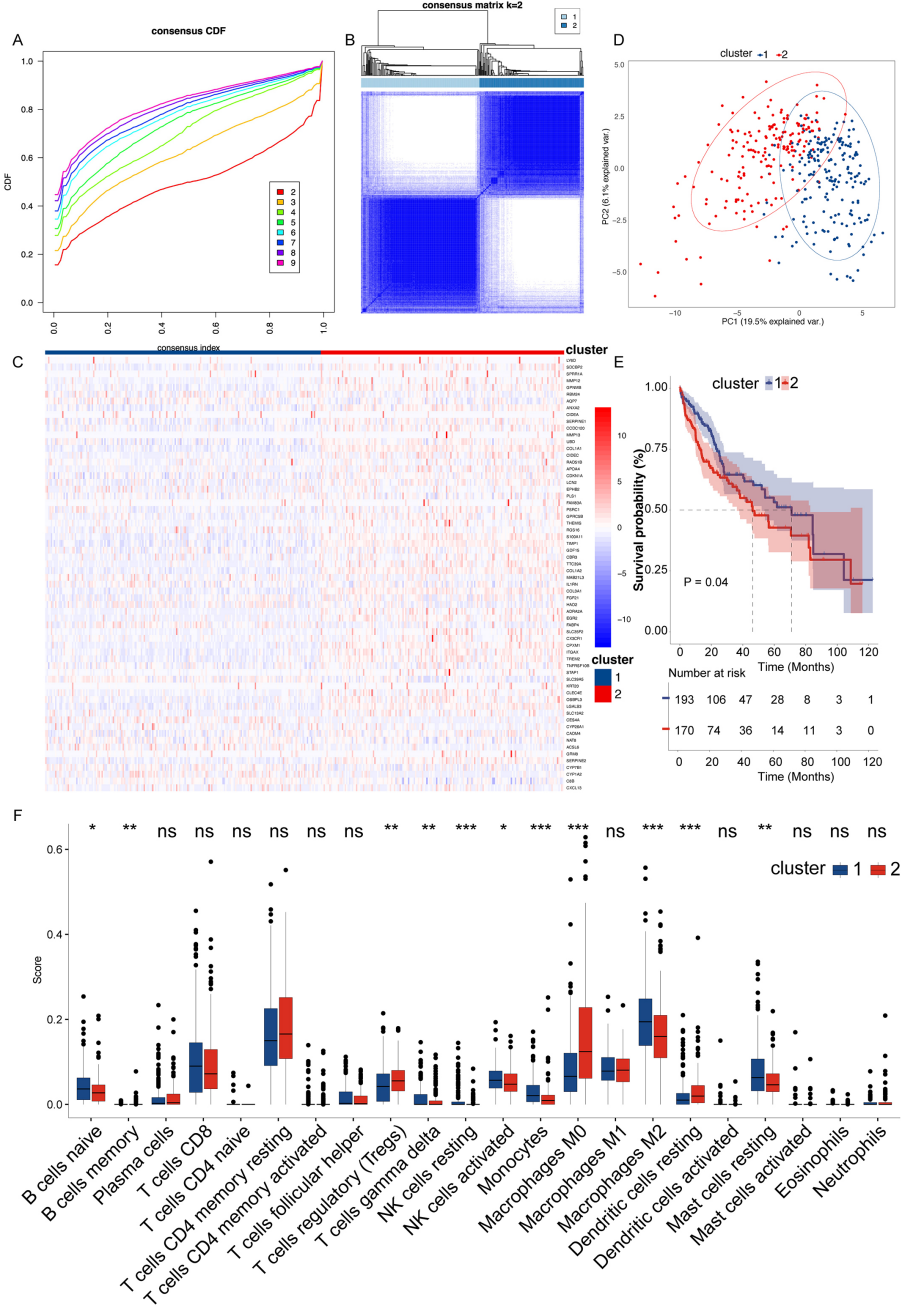
Integrated analysis of gene expression, clustering, and survival in hepatocellular carcinoma. **(A)** Cumulative distribution function (CDF) showing the stability of consensus clustering across 2 to 9 potential clusters, indicating the consistency of data partitioning. **(B)** Consensus matrix for k=2, illustrating the clear separation between the two distinct clusters, highlighted by deep blue (cluster agreement) and white (cluster disagreement) blocks. **(C)** Heatmap of gene expression across the two clusters. Genes are ordered by differential expression between clusters, with red indicating high expression and blue indicating low expression. **(D)** PCA plot delineating the spatial separation between the two clusters based on the first two principal components, capturing 19.5% of the variance, which suggests significant molecular heterogeneity. **(E)** Kaplan-Meier survival curves comparing the overall survival between the two clusters, with shaded areas representing the 95% confidence intervals. Statistical significance is denoted (P = 0.04), suggesting a trend towards different survival outcomes. **(F)** Box plots showing differential immune cell infiltration between the clusters as analyzed by ssGSEA, with immune cell types plotted along the x-axis and enrichment scores on the y-axis. Statistical significance is indicated above each box plot. “*” for P < 0.05; “**” for p < 0.01; “***” for p < 0.001. ns, not significant.

Since NAFLD eventually develops into liver cancer, we therefore examined the role of the hub genes in liver cancer. The consensus clustering analysis (**Figure 3A**) demonstrates that the division into two clusters is the most stable and distinct configuration, as reflected by the sharp increase in CDF values up to k=2. This finding is visually supported by the consensus matrix (**Figure 3B**), which shows a clear segregation between the two clusters. The heatmap provides a detailed view of the differences in central gene expression between clusters, with genes in cluster 2 having higher hub genes expression levels (**Figure 3C**). The PCA plot further confirms the separation between clusters, emphasizing substantial molecular differences that are potentially clinically relevant (**Figure 3D**). Kaplan-Meier survival analysis (**Figure 3E**) showed that liver cancer patients of cluster 2 who highly expressed hub genes had a lower survival rate. Finally, the analysis of immune cell infiltration reveals significant variations in the immune landscapes of the two clusters, which indicates differences in tumor microenvironment and response to immunotherapies (**Figure 3F**). Cluster 2 shows a significant reduction in naive B cells, gamma delta T cells, resting NK cells, activated K cells, monocytes, M2 macrophages, and resting mast cells compared to Cluster 1 (Blue Bars). On the other hand, this cluster exhibits an increase in regulatory T cells (Tregs), M0 macrophages, and resting dendritic cells. The increase in Tregs and M0 macrophages, which are often associated with immunosuppressive activities, suggests an environment that may promote tumor growth and inhibit effective anti-tumor immune responses.

### Positive correlation between hub gene expression and NAS scores highlights genes’ relevance to disease severity

**Figure 4:**
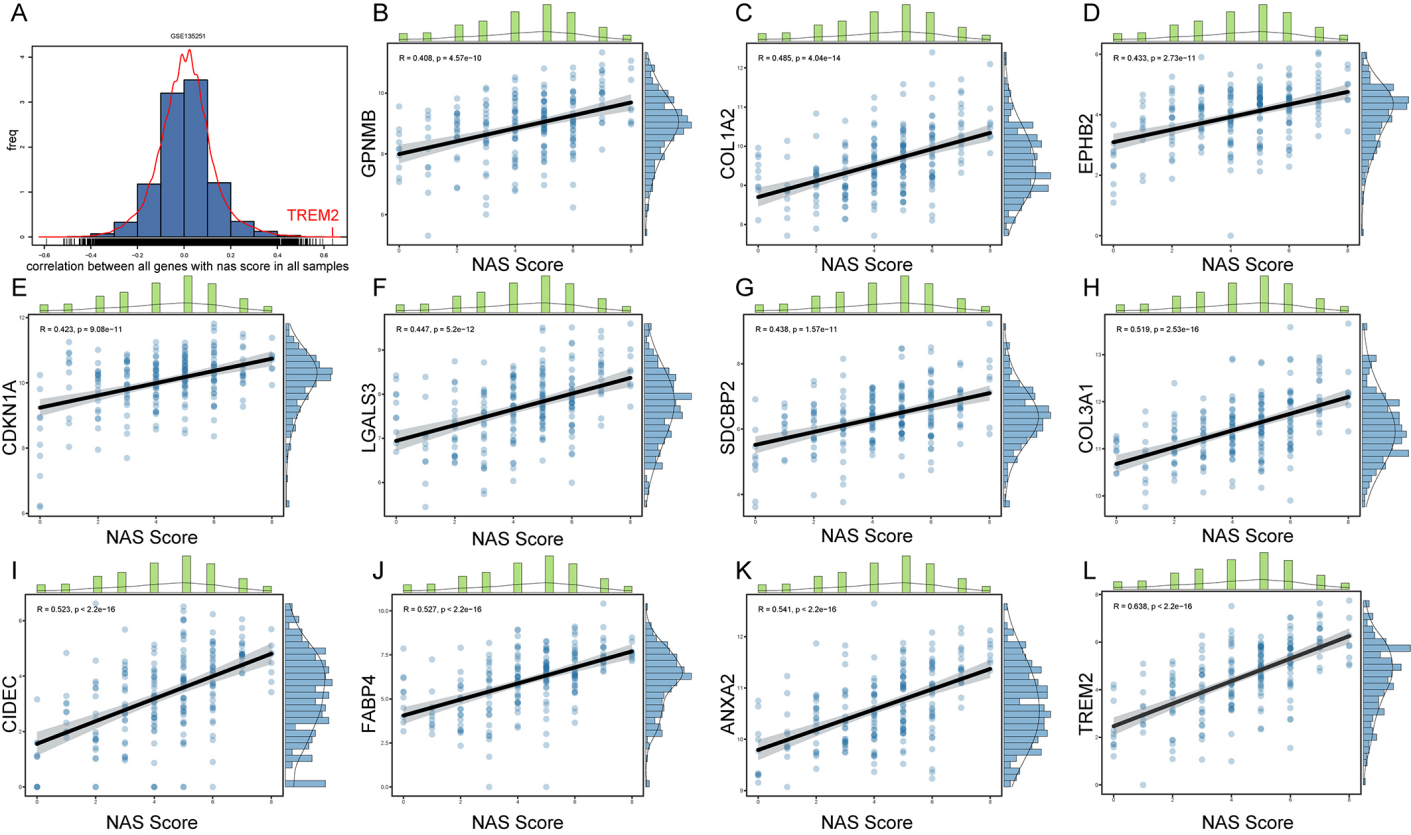
Correlation between gene expression and NAS scores in NAFLD patients. **(A)** Histogram representing the distribution of correlation coefficients between the expression levels of all genes and NAS scores in the GSE135251 dataset. **(B–L)** Scatter plots for genes selected by RRA that have correlation coefficients greater than 0.4 with NAS scores.

To further explore key genes involved in the progression of NASH, we conducted an in-depth analysis using liver sequencing data from patients with NAFLD. We aimed to identify key genes involved in the progression from NASH to HCC based on the following criteria: 1. These genes are positively correlated with the NAS score. 2. Compared to adjacent non-tumorous tissues, these genes are upregulated in cancerous tissues. 3. Patients with high expression of these genes have poorer survival outcomes than those with low expression. 4. These genes are upregulated in NASH-HCC mouse models. GSE135251 dataset includes liver biopsy NAS score (NAFLD Activity Score) ranging from 0 to 8, with 0 referring normal liver histology. We examined the correlation between all genes and NAS scores, especially those obtained by RRA (**Figure 4A**). We identified 11 genes with correlations greater than 0.4 with NAS scores, namely *GPNMB*, *COL1A2*, *EPHB2*, *CDKNN1A*, *LGALS3*, *SDCBP2*, *COL3A1*, *CIDEC*, *FABP4*, *ANXA2*, and *TREM2* (**Figures 4B-L**); 7 genes with correlations between 0.3 and 0.4, namely *CPXM1*, *LCN2*, *ADRA2A*, *SERPINE2*, *GPRC5B*, *UBD*, and *COL1A1* (**Supplementary Figures 2J–P**); 6 genes with correlations between 0.2 and 0.3, namely *OSBPL3*, *ITGAX*, *GDF15*, *CX3CR1*, *TTC39A*, and *SLC15F2* (**Supplementary Figures 2D–I**); and 3 genes with correlations between -0.2 to -0.4, namely *C8B*, *HAO2*, and *AQP7* (**Supplementary Figures 2A–C**).

### Identification of core genes *Trem2*, *Anxa2*, *Gdf15*, and *Ttc39a* as critical markers of poor prognosis in NASH-associated HCC

**Figure 5:**
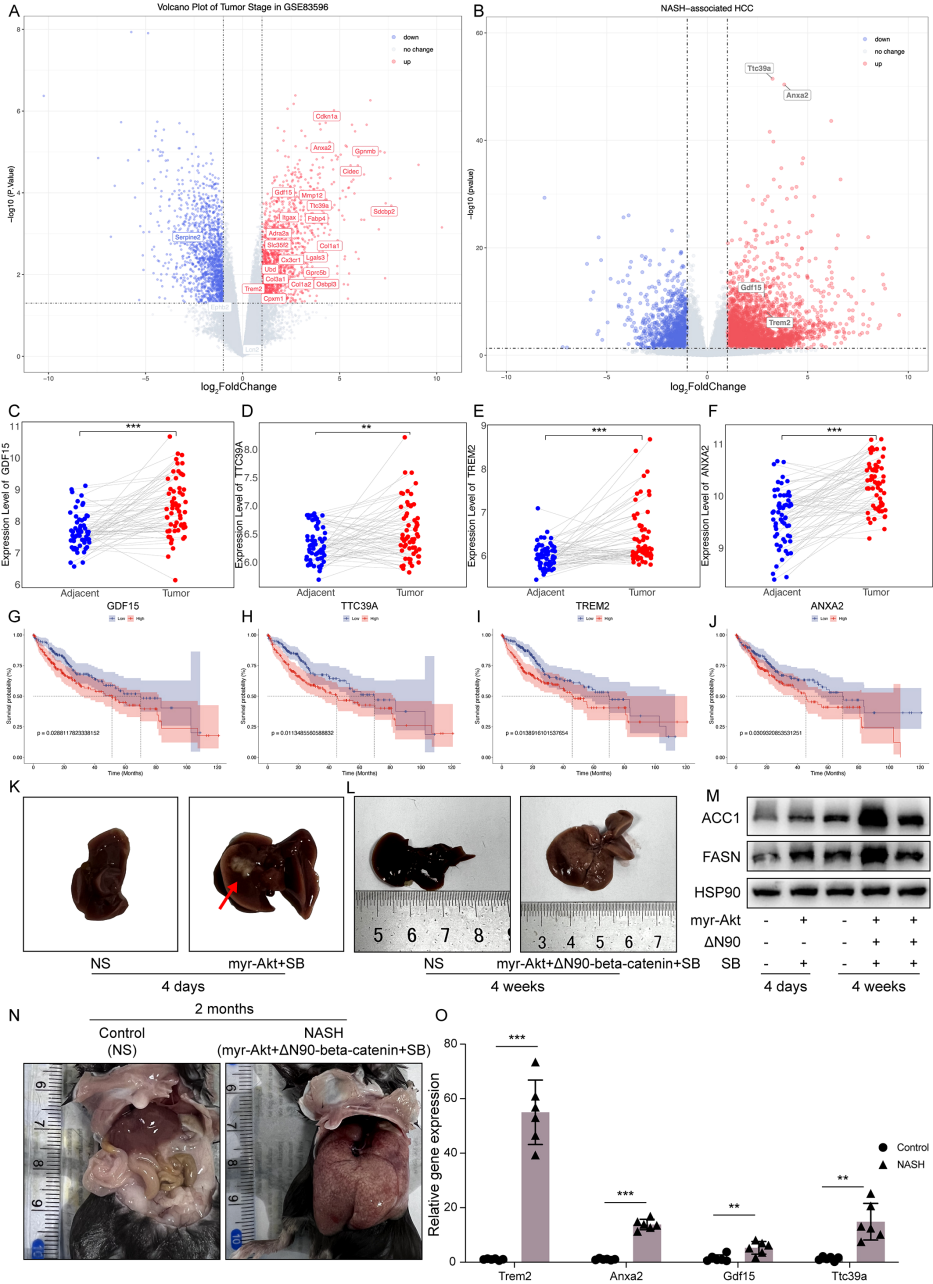
Comprehensive analysis of RRA-selected genes associated with NAS score in NAFLD and prognostic implications in hepatocellular carcinoma. Volcano plots illustrating the expression of genes correlated with NAS score in NASH-associated HCC in GSE83596 **(A)** and GSE246221 **(B)**. Genes significantly upregulated are marked in red, downregulated genes in blue, and non-changing genes in grey. **(C–F)** Paired dot plots showing the expression levels of *GDF15*, *TTC39A*, *TREM2*, and *ANXA2* in adjacent non-tumor and tumor tissues from dataset GSE64041, highlighting significant differences in expression. (**(G–J)**) Kaplan-Meier survival curves for *GDF15*, *TTC39A*, *TREM2*, and *ANXA2* in TCGA liver cancer dataset, categorized into high and low expression groups based on the median expression levels of each gene. **(K)** Mouse liver morphology 4 days after tail vein injection with oncogenic plasmid Akt. **(L)** Mouse liver morphology 4 weeks after tail vein injection with oncogenic plasmids Akt and N90. **(M)** Western blot analysis of ACC1 and FASN proteins in liver samples from mice shown in **(K, L)**. **(N)** Mouse liver morphology 2 months after tail vein injection with oncogenic plasmids myr-Akt and ΔN90-beta-catenin. **(O)** Relative mRNA levels of *Trem2*, *Anxa2*, *Gdf15* and *Ttc39a* in control and NASH mice (n=6). “**” indicates P<0.01, “***” indicates P<0.001.

**Figure 6:**
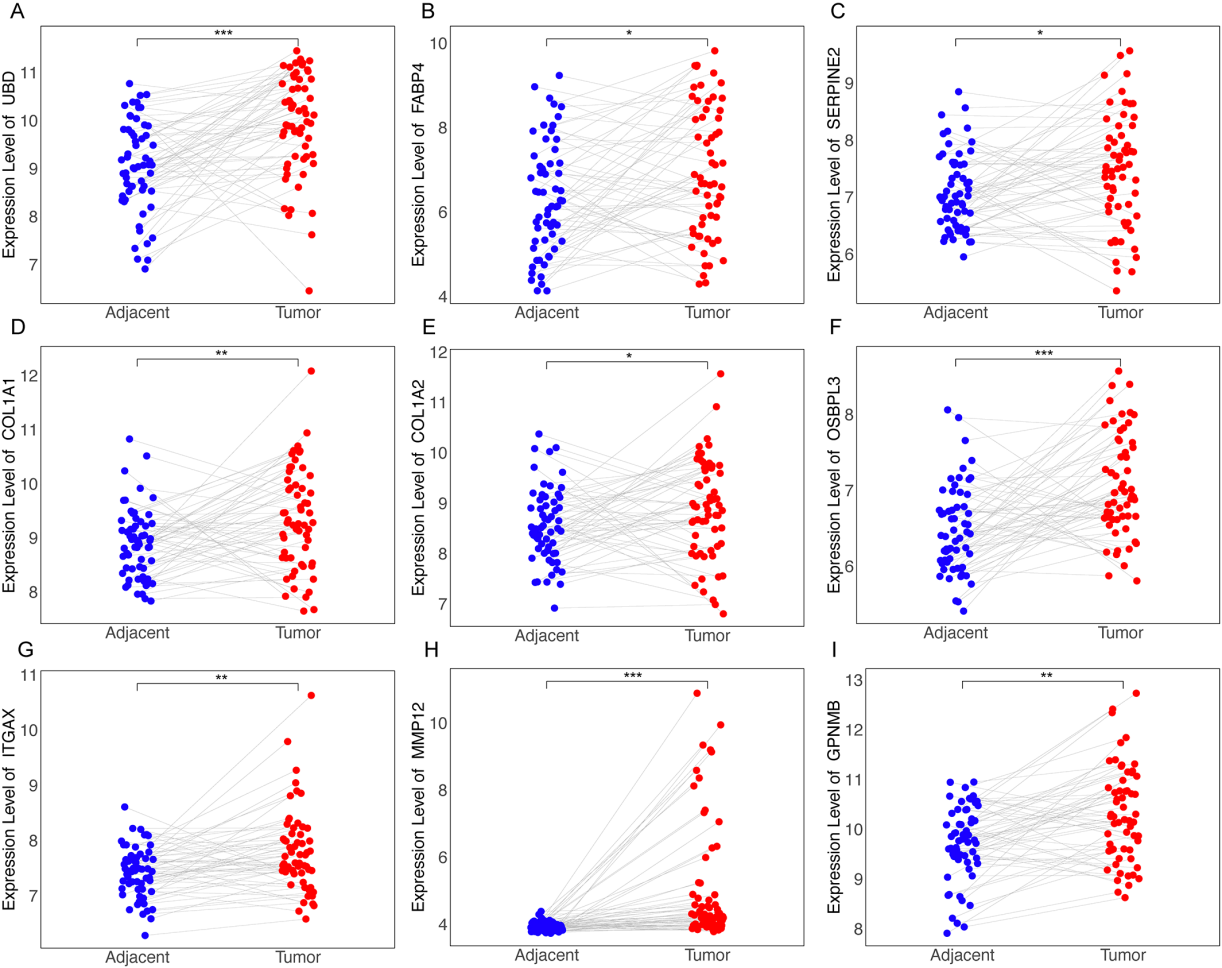
Differential expression of RRA-selected genes in tumor vs. adjacent non-tumor tissues from dataset GSE64041. Dot plots illustrating the expression levels of genes identified through RRA as being upregulated in tumor tissues compared to adjacent non-tumor tissues as *UBD* **(A)**, *FABP4* **(B)**, *SERPINE2* **(C)**, *COL1A1* **(D)**, *COL1A2* **(E)**, *OSBPL3* **(F)**, *ITGAX* **(G)**, *MMP12* **(H)**, *GPNMB* **(I)**. “*” for P < 0.05; “**” for p < 0.01; “***” for p < 0.001.

Forest plots from TCGA hepatocellular carcinoma data show the prognosis of NAS score-related genes whose expression correlates with poor prognosis (**Supplementary Table S1**). Based on the previous criteria for screening key genes for the transition of Non-alcoholic steatohepatitis to HCC, we further screened the 27 genes found to have a positive correlation with the NAS score. We first examined the expression of each of these 27 genes in NASH-induced HCC, and the volcano plot showed that 22 of these genes had elevated expression, which were *Cdkn1a*, *Anxa2*, *Gpnmb*, *Cidec*, *Gdf15*, *Mmp12*, *Ttc39a*, *Itgax*, *Fabp4*, *Sdcbp2*, *Adra2a*, *Slc35f2*, *Col1a1*, *Lgals3*, *Cx3cr1*, *Ubd*, *Gprc5b*, *Col3a1*, *Col1a2*, *Osbpl3*, *Trem2* and *Cpxm1* (**Figure 5A**).We next screened these 22 genes further and found that 13 of them were upregulated in cancer tissues compared to the paraneoplastic ones, which were *GDF15*, *TTC39A*, *TREM2*, *ANXA2*, *UBD*, *FABP4*, *SERPINE2*, *COL1A1*, *COL1A2*, *OSBPL3*, *ITGAX*, *MMP12* and *GPNMB* (**Figures 5C–F**, **6**, **Supplementary Figure 3**). After further screening of these 13 genes, we found that only 4 genes met all criteria for key genes in the transition from NASH to HCC, and they were *GDF15*, *TTC39A*, *TREM2*, and *ANXA2*. These 4 genes were highly expressed in patients with lower survival rates (**Figures 5G-J**). Moreover, we conducted a detailed examination of gene expression in liver tissue from mice with NASH-associated HCC. This model was induced by streptozotocin (STZ) combined with a high-fat diet (HFD), a well-established method for simulating the progression from NASH to HCC, which mimics the human disease pathology closely. Our analysis revealed a significant upregulation in the expression levels of four critical genes: *Trem2*, *Anxa2*, *Ttc39a*, and *Gdf15* (**Figure 5B**). We also analyzed the expression levels of *Trem2*, *Anxa2*, *Ttc39a*, and *Gdf15* across various stages of the disease, including steatosis (**Supplementary Figure 4A**), NASH (**Supplementary Figure 4B**), and fibrosis (**Supplementary Figure 4C**). Remarkably, we observed that these genes were not only upregulated in the NASH-associated HCC stage but also showed increased expression at earlier disease stages. The consistent upregulation of *Trem2*, *Anxa2*, *Ttc39a*, and *Gdf15* across all stages of liver disease—from steatosis and NASH to fibrosis—suggests that these genes play a pivotal role in the progression of NAFLD.

To better simulate the transition from NASH to HCC, we administered oncogenic plasmids AKT and N90 and the sleeping beauty transposon (SB) via tail vein injection. Four days post-injection, fat accumulation was observed in the mouse liver (**Figure 5K**); by four weeks, severe liver damage occurred, and ACC1 and FASN protein levels significantly increased (**Figures 5L, M**). This confirms the successful creation of the NASH mouse model. Two months after injecting the AKT and N90 plasmids, there were significant morphological changes and severe damage in the liver, with evident fatty liver (**Figure 5N**). The mRNA levels of the four genes, *GDF15*, *TTC39A*, *TREM2*, and *ANXA2*, were significantly elevated, as shown in **Figure 5O**, indicating that these genes may play a crucial role in the transition from NASH to HCC.

### Core genes are associated with immune responses in the transition from NASH to HCC

Previous results showed significant differences in immune infiltration among liver cancer patients grouped by hub genes (**Figure 3F**). We further analyzed the role of key genes in immune infiltration in NASH-associated liver cancer patients using GSEA on the GSE164760 dataset. Specifically, there is a downregulation of Th1 and Th2 cell differentiation pathways in cancerous tissues, which may weaken immune surveillance of tumor cells, as Th1 cells promote the attack and elimination of tumor cells (**Supplementary Figure 5A**). Concurrently, key pathways such as Endocytosis, Necroptosis, Chemical Carcinogenesis – Reactive Oxygen Species, Cell Cycle, and Non-Alcoholic Fatty Liver Disease are upregulated, reflecting their pivotal roles in promoting disease progression and tumor development (**Supplementary Figures 5B–F**). Violin plots using the ESTIMATE algorithm confirmed higher Tumor Microenvironment (TME) scores in non-cancerous than in cancerous tissues of NASH-associated HCC patients (**Supplementary Figure 5G**), indicating more pronounced stromal and immune components in the former. Additionally, scatter plots (**Supplementary Figure 6**) revealed a positive correlation between *TREM2* and *ANXA2* with both the StromalScore and ImmuneScore, suggesting their roles in modulating the tumor microenvironment. ESTIMATEScore, ImmuneScore, and StromalScore were higher in patients with high expression of *TREM2* and *ANXA2*, whereas these scores did not differ in patients with high expression of *GDF15* and *TTC39A* (**Supplementary Figure 7**).

### Elevated expression of core genes *Trem2*, *Anxa2*, *Gdf15*, and *Ttc39a* in single-cell analyses of NASH mouse models

**Figure 7:**
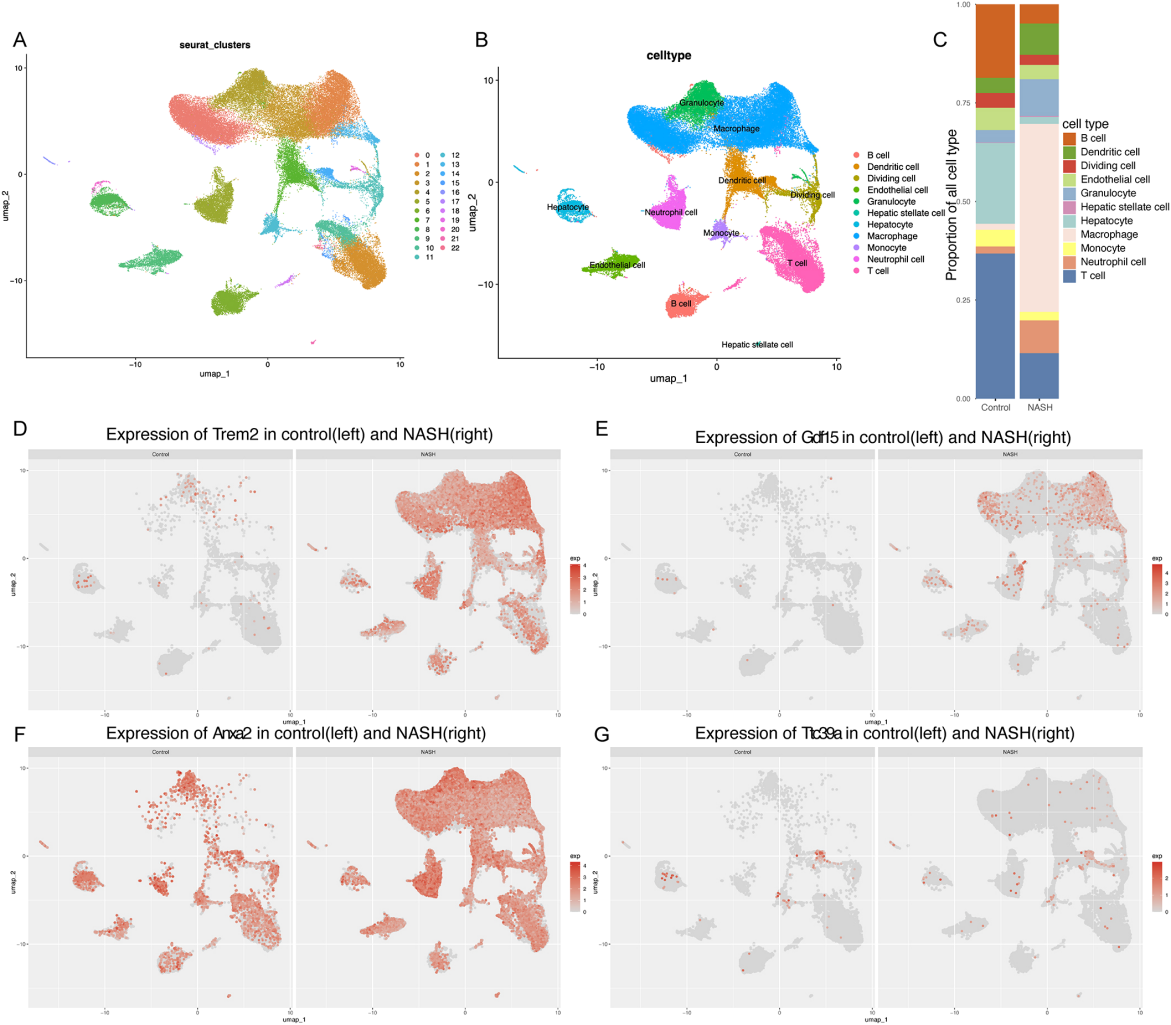
Single-cell analysis of gene expression in the NASH liver tissues. **(A, B)** UMAP dimensionality reduction plot displaying the diverse cellular landscape in the dataset GSE216836, with each cell type color-coded for identification.**(C)** Proportion of all cell types in Control and NASH. **(D-G)** Feature plots for *Trem2* **(D)**, *Gdf15* **(E)**, *Anxa2* **(F)**, and *Ttc39a* **(G)** in control group and NASH group.

To further explore the possible role of *GDF15*, *TTC39A*, *TREM2*, and *ANXA2* in the progression of NAFLD to HCC, we used the single-cell dataset GSE216836 to examine the expression of these genes. The UMAP plot effectively segregates various cell types, providing a comprehensive view of the cellular heterogeneity (**Figures 7A, B**). We found that the proportions of various cell types in control and NASH liver tissue samples. Notably, the proportion of macrophages significantly increases in the NASH samples compared to the control, while T cells and B cells show a relative decrease in their proportions(**Figure 7C**). We annotated each cell cluster identified in our study using the CellMarker database, a comprehensive resource for cell markers across various tissues and organisms(**Supplementary Figure 8**).

**Figure 8:**
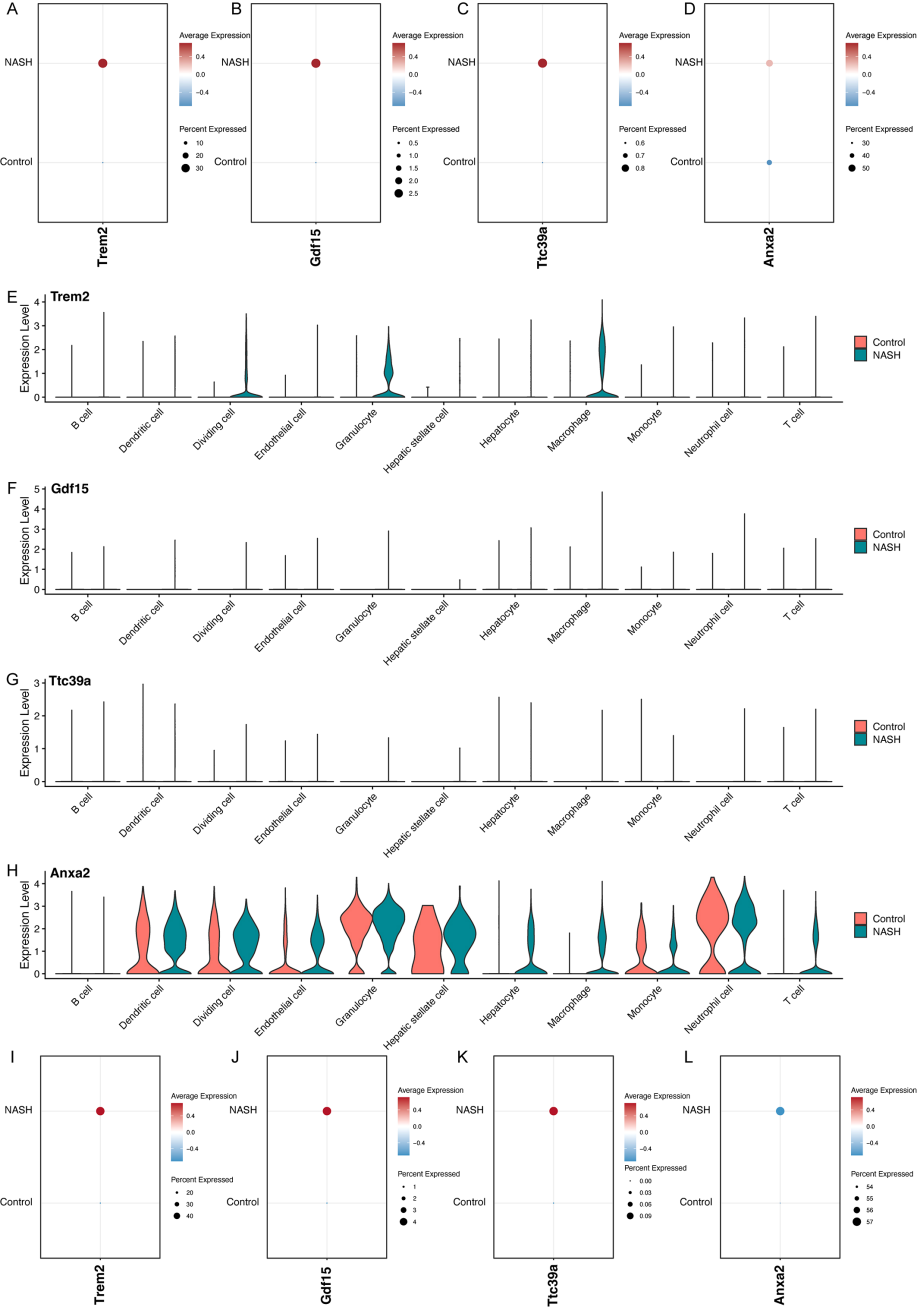
Analysis of Gene Expression in Control and NASH Samples Across Different Cell Types. Dot plots showing the overall expression levels of *Trem2* **(A)**, *Gdf15* **(B)**, *Ttc39a* **(C)**, and *Anxa2* **(D)** in control and NASH samples. Violin plots showing the expression levels of *Trem2* **(E)**, *Gdf15* **(F)**, *Ttc39a* **(G)** and *Anxa2* **(H)** across various cell types in control and NASH. Dot plots showing the expression levels of *Trem2* **(I)**, *Gdf15* **(J)**, *Ttc39a* **(K)**, and *Anxa2* **(L)** in macrophage in control and NASH.

**Figure 9:**
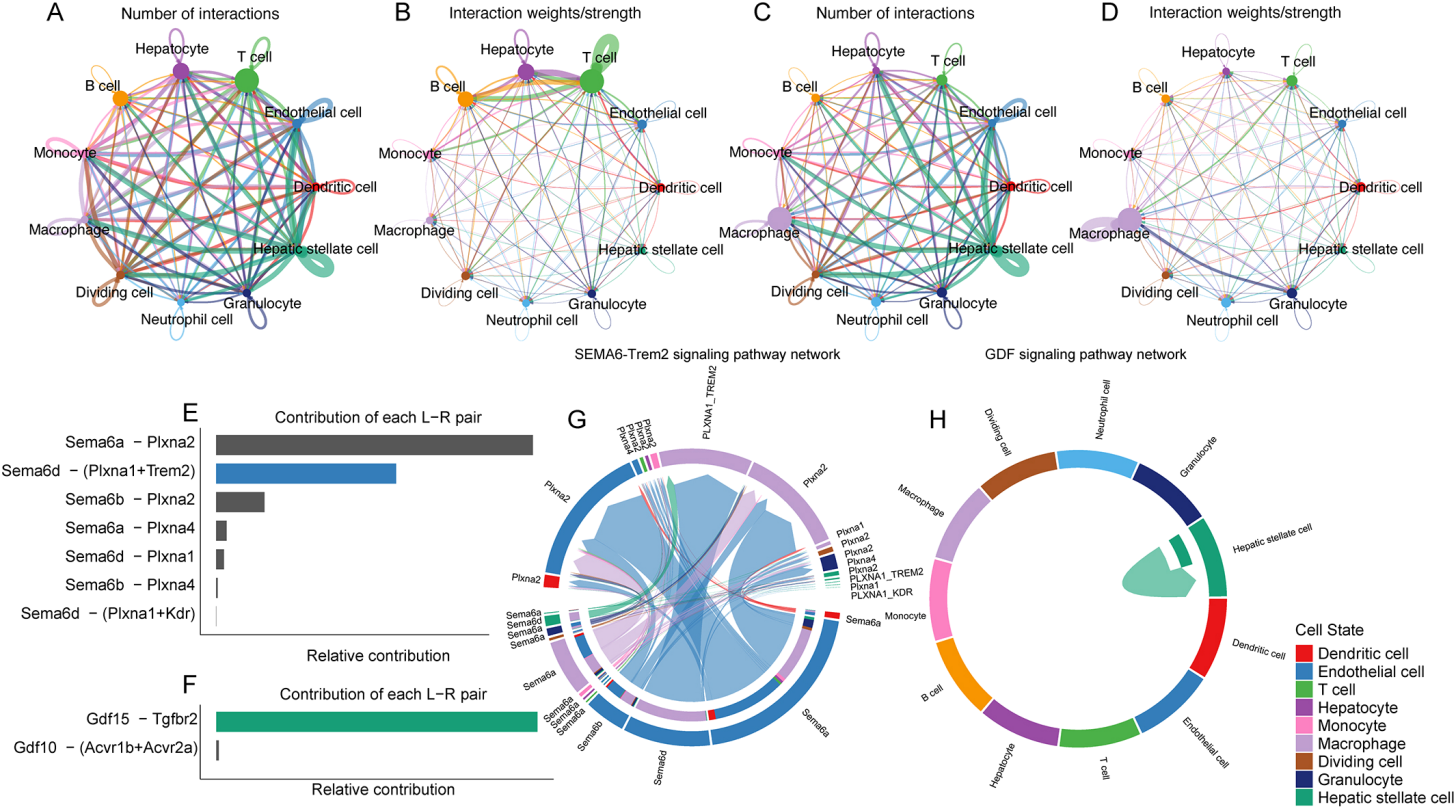
Cellular communication networks in control and NASH conditions through single-cell transcriptomic analysis of dataset GSE216836. **(A)** Network diagram showing the number of interactions between various cell types in the control group. Each node represents a cell type, and the thickness of the connecting lines indicates the number of interactions. **(B)** Network diagram illustrating the interaction strength between different cell types in the control group, with thicker lines representing stronger interactions. **(C)** Network diagram showing the number of interactions between various cell types in the NASH group. **(D)** Network diagram illustrating the interaction strength between different cell types in the NASH group, with thicker lines representing stronger interactions. **(E)** Bar chart showing the relative contribution of different ligand-receptor pairs in the Sema6 signaling pathway. Notably, the interaction between Trem2 and Plxna1 is highlighted. **(F)** Bar chart detailing the contributions of ligand-receptor pairs in the Gdf signaling pathway, focusing on the interaction between Gdf15 and Tgfbr2. **(G)** Chord diagram illustrating the SEMA6-Trem2 signaling network across different cell types, highlighting the complexity and connectivity of this pathway. **(H)** Circular plot showing the distribution of the GDF signaling pathway across various cell types.

Overall, the feature plots and dot plots indicate an increased expression of the four genes *Trem2*, *Anxa2*, *Ttc39a*, and *Gdf15* in NASH (**Figures 7D–G**, **8A-D**). Specifically, there is a noticeable increase in the expression levels of these genes in macrophages within NASH (**Figures 8E-L**). The analysis of cellular communication within the control and NASH conditions revealed distinct patterns of intercellular interactions. In the control condition, the network diagrams (**Figures 9A, B**) displayed a balanced communication pattern among various cell types, with endothelial cells, hepatocytes, and macrophages showing prominent interactions. In contrast, the NASH condition (**Figures 9C, D**) exhibited a significant increase in communication activities, especially involving inflammatory cells such as dendritic cells and macrophages, indicating heightened immune response and cellular stress. The Sema6 signaling pathway analysis (**Figure 9E**) identified key interactions, particularly between Trem2 and Plxna1, suggesting their pivotal roles in cellular navigation and immune regulation. Similarly, the GDF signaling pathway (**Figure 9F**) analysis verified the interaction between Gdf15 and Tgfbr2, crucial for tissue repair and fibrosis, which was often exacerbated in NASH. The complex connections of SEMA6-Trem2 in endothelial cells and macrophage networks (**Figure 9G**) and the distribution of the GDF pathway in hepatic stellate cells (**Figure 9H**) highlight the complex network of signaling events that coordinate cellular responses in liver disease. This analysis emphasizes the intricate interplay between gene expression and the immune landscape, providing insights into potential therapeutic targets and prognostic indicators in NASH-associated HCC.

### Prognostic significance of elevated *Trem2*, *Anxa2*, *Gdf15*, and *Ttc39a* expression correlates with poor outcomes and higher TP53 mutation rates in cancer patients

**Figure 10:**
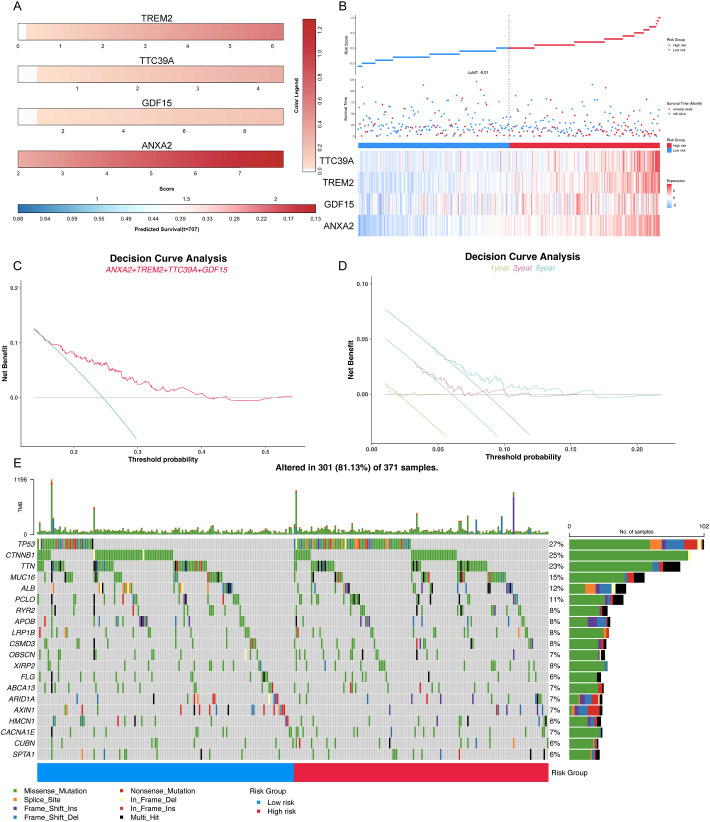
Comprehensive analysis of key prognostic genes *ANXA2*, *TREM2*, *GDF15*, and *TTC39A* in HCC. **(A)** Color striped plots representing the expression levels of the four genes across HCC samples. **(B)** The risk factor association diagram showing risk score distribution, survival status, and the expression of *ANXA2*, *TREM2*, *GDF15*, and *TTC39A*. **(C)** Decision curve analysis (DCA) for overall survival based on the expression levels of *ANXA2*, *TREM2*, *GDF15*, and *TTC39A*, demonstrating the clinical utility of these genes as prognostic biomarkers in HCC. **(D)** Year-specific DCA curves for 1-year, 3-year, and 5-year survival rates, illustrating the predictive power of the key genes at various time points. **(E)** Oncoplot visualizing the mutation status of the high-risk and low-risk groups, highlighting differences in genetic alterations that might influence prognosis.

Clinical prognostic analysis of the core genes *TREM2*, *TTC39A*, *GDF15*, and *ANXA2* demonstrated that their expression levels were significantly correlated with the survival outcomes of patients diagnosed with HCC. Nomogram of the core genes showed varying expression levels across different patient samples, with higher expression of these genes correlating with poorer predicted survival probabilities (**Figure 10A**). **Figure 10B** illustrates the survival status, risk score distribution, and expression levels of the core genes. The mortality rate in the high-risk group is significantly higher than in the low-risk group, and the expression levels of the core genes are elevated in the high-risk group compared to the low-risk group. Decision curve analysis revealed a significant net benefit across various practical threshold probabilities for clinical decision-making, highlighting the robust potential of *ANXA2*, *TREM2*, *GDF15*, and *TTC39A* as biomarkers for predicting overall survival in patients with HCC (**Figure 10C**). The year-specific DCA curves further validated the efficacy of these biomarkers in forecasting short-term and long-term survival, essential for personalized treatment planning (**Figure 10D**). Patients were stratified into high-risk and low-risk groups based on gene expression profiles. High-risk patients, identified by the higher expression of the gene signature, demonstrated a more extensive and varied mutation pattern compared to the low-risk group (**Figure 10E**). The mutations of key genes in 371 LIHC samples were visualized using oncoplot, and the results showed that the high-risk group exhibited higher TP53 mutations (**Figure 10E**). This stratification validated the potential link between mutation burden and poorer clinical outcomes, suggesting that patients in the high-risk group were prone to experience more aggressive disease progression. Overall, these results suggest that the signature composed of these four core genes may serve as an important prognostic indicator for NASH-associated HCC.

### Reduced drug sensitivity in patients with high expression of core genes *TREM2*, *ANXA2*, *GDF15*, and *TTC39A*

**Figure 11:**
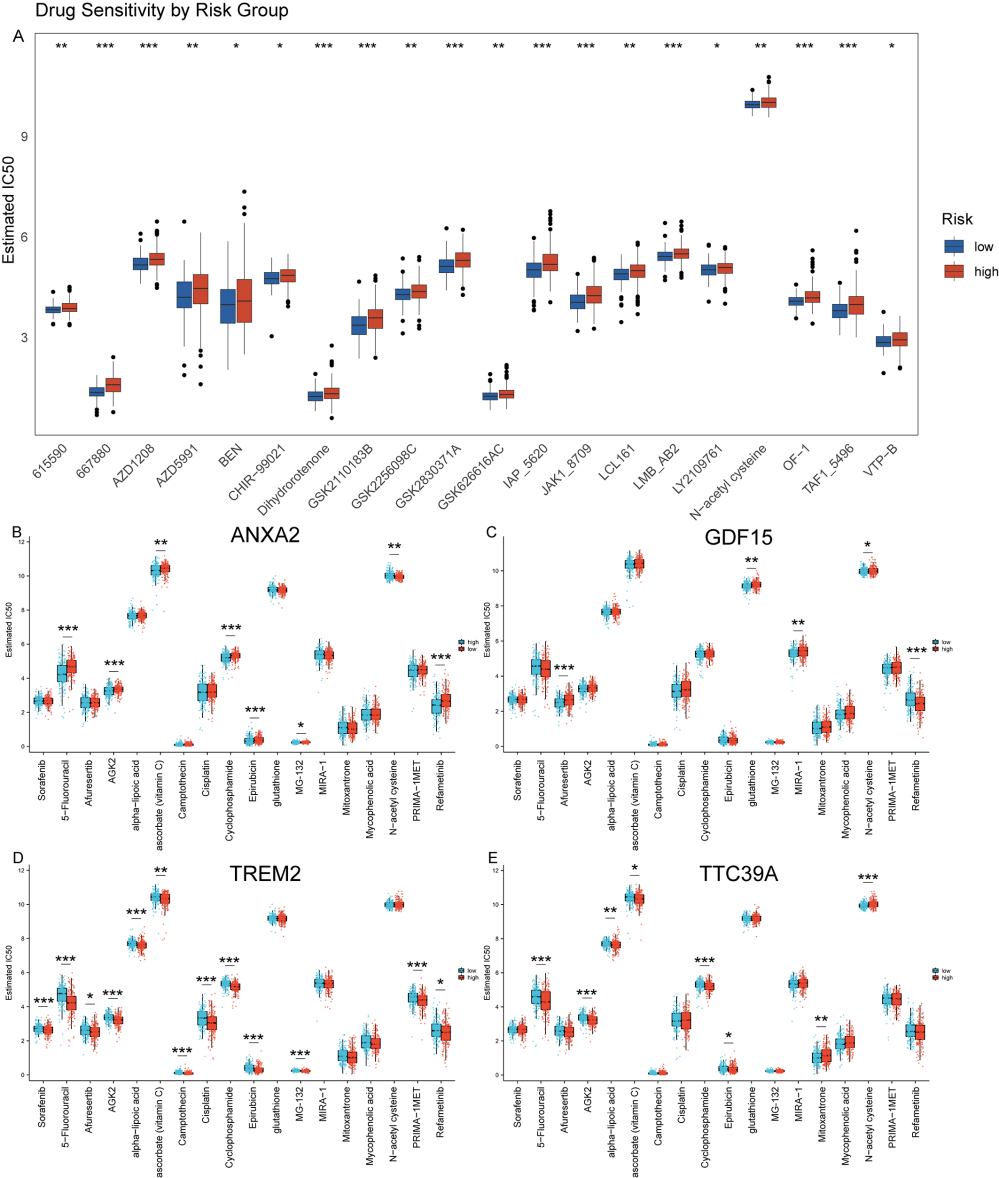
Prediction of drug sensitivity using GDSC database. **(A)** Drug sensitivity analysis using the GDSC database comparing the response of high and low-risk groups to various chemotherapeutic agents, demonstrating differential susceptibility to treatment based on risk stratification. **(B–E)** Analysis of drug sensitivity in HCC based on expression levels of *ANXA2*, *GDF15*, *TREM2*, and *TTC39A* using the GDSC Database. “*” for P < 0.05; “**” for p < 0.01; “***” for p < 0.001.

We used GDSC database to predict the chemotherapy response of the common chemotherapy agents between the two groups. The drug sensitivity analysis demonstrates that differential drug responsiveness is associated with risk classification based on these four core genes (**Figure 11A**). We found that high-risk patients were insensitive to the following compounds: 615590, 667880, AZD1208, AZD5991, BEN, CHIR-99021, Dihydrorotenone, GSK2110183B, GSK2256098C, GSK2830371A. GSK626616AC, IAP_5620, JAK1_8709, LCL161, LMB_AB2, LY2109761, N-acetyl cysteine, OF-1, TAF1_5496, VTP-B (**Figure 11A**). Additionally, we assessed the sensitivity to some common anticancer drugs. Generally speaking, HCC patients with NASH exhibit higher sensitivity to the following drugs, as their IC50 values are close to 0: Camptothecin, Epirubicin, MG-132, Mitoxantrone, and Mycophenolic acid. However, for most other drugs, there are higher IC50 values, such as: Sorafenib, 5-Fluorouracil, Afuresertib, AGK2, alpha-lipoic acid, ascorbate (vitamin C), Cisplatin, Cyclophosphamide, glutathione, MIRA-1, N-acetyl cysteine, PRIMA-1MET, and Refametinib (**Figures 11B–E**). Moreover, we further analysis the drug sensitivity individually based on the four key genes expression level (**Figures 11B–E**). The groups were categorized based on the median expression level of the genes. Patients with gene expression levels above the median are classified into the high group, while those with expression levels below the median are categorized into the low group. Patients with high expression of *ANXA2* are insensitive to 5-Fluorouracil, Cyclophosphamide, Epirubicin, MG-132, and Refametinib but are sensitive to Sorafenib, Afuresertib, AGK2, Ascorbate (Vitamin C), and N-acetyl cysteine (NAC) (**Figure 11B**). Patients with high expression of *GDF15* show insensitivity to Afuresertib, whereas they are sensitive to 5-Fluorouracil, Glutathione, MIRA-1, NAC, and Refametinib (**Figure 11C**). Patients with high expression of *TREM2* are sensitive to Sorafenib, 5-Fluorouracil, Afuresertib, AGK2, Alpha-lipoic acid, Vitamin C, Camptothecin, Cisplatin, Cyclophosphamide, Epirubicin, MG-132, PRIMA-1MET and Refametinib (**Figure 11D**). Patients with high expression of *TTC39A* are insensitive to Mitoxantrone and NAC, but are sensitive to Sorafenib 5-Fluorouracil, AGK2, Vitamin C, Cyclophosphamide and Epirubicin (**Figure 11E**). This finding suggests that these genes not only serve as prognostic biomarkers but may also guide therapeutic decision-making, particularly in selecting more effective personalized treatment regimens for HCC patients.

### Diminished immunotherapy efficacy in patients with high expression of core genes *TREM2*, *ANXA2*, *GDF15*, and *TTC39A*

**Figure 12:**
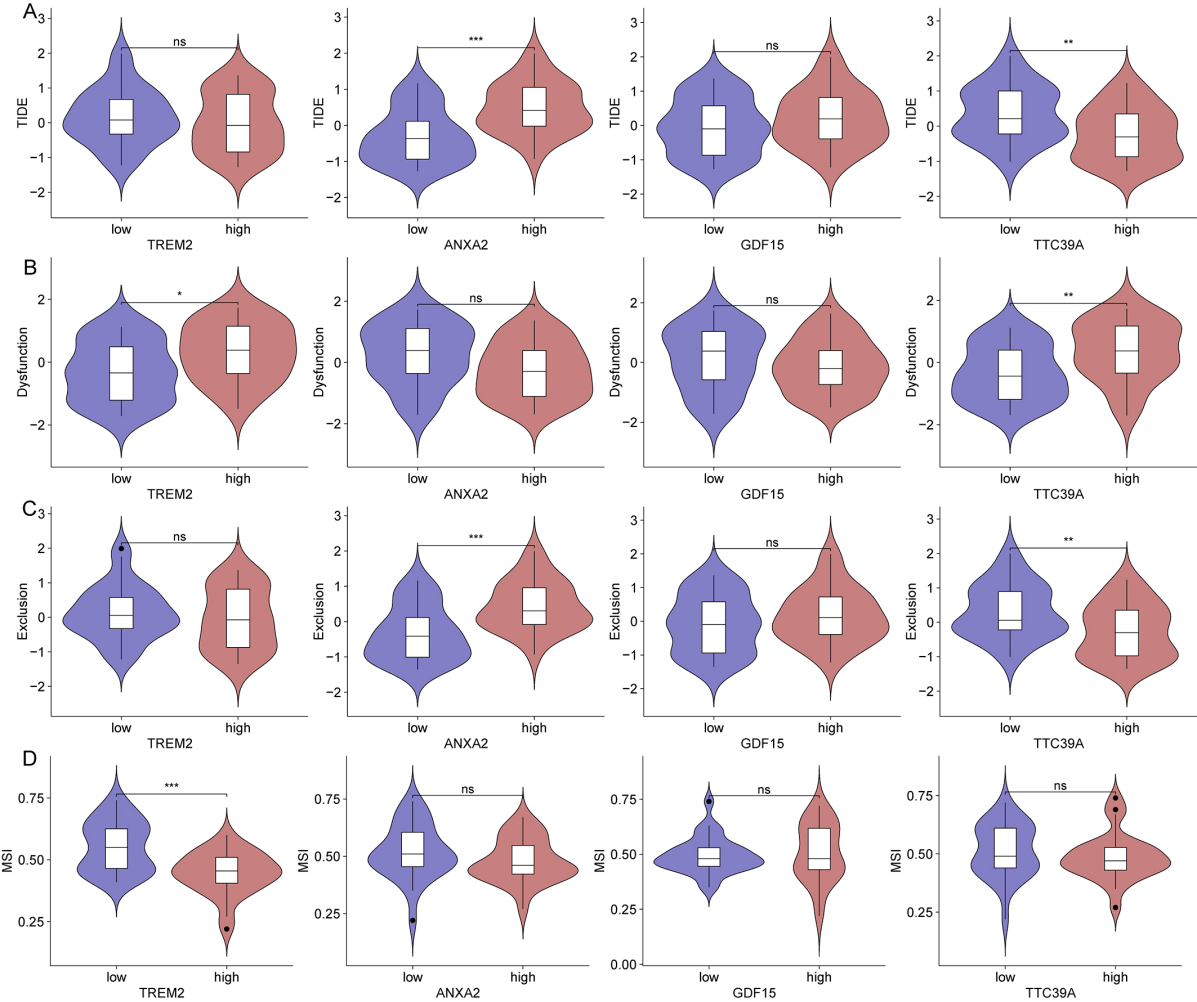
Impact of gene expression on immune metrics in NASH-associated hepatocellular carcinoma based on dataset GSE164760. **(A)** Violin plots showing TIDE scores distributed across high and low expression groups of *TREM2*, *ANXA2*, *GDF15*, and *TTC39A* in NASH-associated hepatocellular carcinoma. **(B)** Violin plots depicting Dysfunction scores for high and low expression groups of the core genes. **(C)** Violin plots for Exclusion scores across high and low expression groups of these genes. **(D)** MSI scores presented in violin plots comparing high versus low expression groups of the genes. “*”: p < 0.05; “**”: p < 0.01; “***”: p < 0.001. ns, not significant.

We then analyzed the relationship between the expression of key genes and various immune metrics in hepatocellular carcinoma associated with NASH. Patients with high *ANXA2* expression exhibit higher TIDE and Exclusion scores (**Figures 12A, C**). Patients with high *TREM2* and *TTC39A* expression show higher Dysfunction scores (**Figure 12B**). Additionally, patients with high TREM2 expression have lower MSI scores (**Figure 12D**), suggesting these genes may contribute to immune evasion mechanisms in the tumor environment. Notably, patients who respond to immunotherapy have lower *ANXA2* expression levels (**Supplementary Figure 9A**). Patients with high *TREM2* and *TTC39A* expression show higher levels of CD274, while those with high *ANXA2* expression have lower *CD274* levels (**Supplementary Figure 9B**). Additionally, patients with high *GDF15* expression have lower *CD8* expression levels (**Supplementary Figure 9C**). These findings emphasize the potential negative impact of the expression levels of these genes on the efficacy of immunotherapy in NASH-associated HCC. High expression of *ANXA2*, *TREM2*, *TTC39A*, and *GDF15* may lead to a more suppressive tumor microenvironment and enhanced immune escape, thereby reducing the efficacy of immunotherapy.

## Discussion

The exploration of the transition from NASH to HCC remains a critical area of research due to the increasing prevalence of NAFLD and its potential to develop into more severe forms. Our study aimed to identify key genes that could serve as biomarkers and therapeutic targets, enhancing our understanding of disease progression and providing new avenues for clinical intervention. Our findings underline the complex interplay of genetic and epigenetic modifications that drive the progression from NASH to HCC. The identified genes, including *TREM2*, *GDF15*, *TTC39A*, and *ANXA2*, have shown significant roles not only in the pathological process but also in influencing the prognosis and therapeutic response of HCC. We utilized a Hydrodynamic Transfection to simulate the transition from NASH to HCC. This technique involves the rapid injection of a large volume of DNA solution into the mouse’s tail vein, generating hydrodynamic pressure sufficient to temporarily disrupt the endothelial barrier of the liver capillaries, allowing DNA to enter the hepatocytes (27). During this process, the “Sleeping Beauty” transposon system is used to promote somatic integration of DNA, ensuring long-term gene expression. This transposon system can recognize and bind to specific inverted repeat sequences at both ends of the DNA sequence, then cut and paste the DNA from one location to another. Thus, the carcinogenic genes carried by the plasmid DNA are stably integrated into the host genome, allowing for long-term expression in liver cells, ultimately inducing liver cancer formation. This modeling method effectively simulates the progression of the disease *in vivo*, from the initial stage of steatosis, through subsequent NASH, to the final stage of developing into HCC (**Figures 5K-O**). This aligns with current literature that describes a multifactorial progression mechanism involving inflammatory pathways, metabolic dysfunction, and immune system interactions.

*TREM2* is a transmembrane receptor expressed on myeloid cells, integral to the immune system’s response to cancerous growths (28). It acts as a significant immunological and prognostic biomarker across various cancer types, including HCC (29). The expression of *TREM2* varies across different cancers and generally correlates with poor prognosis when upregulated (30), indicating its potential as a target for therapeutic intervention (31). *TREM2*’s role extends beyond traditional immune responses, impacting TME dynamics significantly (32). In the context of HCC, *TREM2* expression influences the infiltration and function of tumor-associated macrophages (TAMs) and myeloid-derived suppressor cells (MDSCs), which are pivotal in mediating tumor immunity and sculpting the inflammatory landscape of the TME. Furthermore, TREM2 influences key signaling pathways such as the Wnt/β-catenin and PI3K/Akt pathways, which are crucial in oncogenesis and tumor progression (33). Notably, the relationship between *TREM2* expression and various clinical phenotypes, such as tumor stage and patient survival, reinforces its potential utility in clinical assessments and personalized treatment planning. In HCC specifically, *TREM2*’s modulation of macrophage activity within the liver can either promote a pro-tumorigenic environment conducive to cancer progression or enhance immune checkpoint blockade therapy, depending on its expression levels and the context of other immune modulators within the TME (34).

In our study, we found that liver cancer patients with high *TREM2* expression have a lower survival rate (**Figure 5I**). Additionally, *TREM2* has the highest correlation with NAS score among all genes (**Figure 4A**), and it is closely associated with immune infiltration (**Supplementary Figure 6A**). Thus, targeting *TREM2* or modulating its pathway could provide a strategic point of intervention to alter the immune landscape in HCC, potentially improving patient outcomes in immunotherapy and other therapeutic approaches.

*TTC39A-AS1* was shown to function as a competing endogenous RNA, sponging miR-483-3p to upregulate MTA2 in breast cancer, thereby promoting tumorigenicity (35). This mechanism of action prompts a potential role of *TTC39A* in modulating gene expression through noncoding RNAs in liver disease as well. Research into the expression and roles of *TTC39A* and its associated noncoding RNAs in liver disease could help clarify their potential as biomarkers or therapeutic targets. Understanding how these molecules interact with miRNAs and other components of the cellular machinery in the liver will be crucial. Such studies could lead to novel therapeutic strategies that specifically target the molecular pathways influenced by *TTC39A* and its noncoding RNAs, potentially halting or reversing the progression of liver diseases. The regulatory activities of *TTC39A* and its associated noncoding RNAs, as illustrated in breast cancer research, provide a compelling model that could be applicable to liver diseases. In this study, we found a positive correlation between *TTC39A* and the ImmuneScore (**Supplementary Figure 6D**). Additionally, using the TIDE database for predictive analysis, we discovered that patients with high *TTC39A* expression exhibit greater T-cell dysfunction (**Figure 12B**). This may also be related to the transition from NASH to HCC. Investigating these mechanisms in the context of NAFLD and HCC could uncover new molecular targets for therapy and deepen our understanding of liver disease progression.

*ANXA2* is an important member of the annexin family and is expressed on the surface of various tumor cells. *ANXA2* has multiple functions, including involvement in endocytosis, cytokinesis, actin remodeling, signal transduction, protein assembly, mRNA transport, and DNA repair (36, 37). Recent studies highlight the critical role of *ANXA2* as a pivotal regulator in the pathogenesis and progression of various liver diseases, including NAFLD and HCC (38, 39). *ANXA2* influences liver fibrosis through its interactions with cellular pathways that regulate extracellular matrix remodeling. Specifically, the ANXA2-Notch regulatory loop plays a crucial role in promoting liver fibrosis in NAFLD by modulating osteopontin expression. In the context of hepatocarcinogenesis, *ANXA2* is significantly upregulated and plays a role in the mesenchymal stem cell-mediated progression of liver cancer. Mesenchymal stem cells enhance the malignant characteristics of HCC cells, partly through the lncRNA-MUF interaction with *ANXA2*, which activates Wnt/β-catenin signaling and promotes epithelial–mesenchymal transition (38). Machine learning analyses have identified *ANXA2* among the ferroptosis-related genes as potential diagnostic biomarkers for NAFLD, suggesting its involvement in the oxidative stress response and its potential utility in early diagnosis (39). Given its central role in pathways crucial to liver health, targeting *ANXA2* represents a novel approach to treat liver diseases (40). Its interactions with key signaling pathways provide promising therapeutic targets, especially in preventing the progression from NAFLD to NASH and HCC. In this study, *ANXA2* expression showed a strong correlation with the NAS score (**Figure 4K**), and among the four core genes, *ANXA2* plays the most significant role in the prognosis of HCC patients (**Figure 10A**). Additionally, patients with high *ANXA2* expression exhibited more immune exclusion (**Figure 12C**). *ANXA2* is not only involved in liver fibrosis and hepatocarcinogenesis but also shows potential as a diagnostic biomarker, making it a significant focus in current liver disease research. Continued investigation into the molecular mechanisms of *ANXA2* will enhance our understanding of its roles in liver disease and support the development of targeted therapeutic strategies.

*GDF15*, a stress-responsive cytokine, plays a critical role in liver disease progression, especially in NAFLD and fibrosis. It is intricately involved in regulating inflammation and cellular stress responses, which are pivotal factors in the advancement of these conditions (41). *GDF15* levels have been positively associated with the severity of fibrosis in patients with biopsy-proven NAFLD, indicating its potential as a biomarker for the progression of liver fibrosis (42). Elevated *GDF15* levels correlate with advanced stages of fibrosis, suggesting that *GDF15* could be used to identify patients at higher risk of progressing to severe liver diseases, including cirrhosis and hepatocellular carcinoma (42, 43). Furthermore, the role of *GDF15* in liver disease extends beyond a simple marker of disease severity. It has been demonstrated to influence cellular processes such as apoptosis and inflammation by interacting with multiple signaling pathways, notably the SMAD pathways, which play a critical role in fibrogenesis (41). Specifically, *GDF15* has been found to promote the activation of hepatic stellate cells, a key event in the development of liver fibrosis, by enhancing the expression of fibrotic markers and the phosphorylation of SMAD2 and SMAD3 proteins (43). In this study, we found that *GDF15* in HSCs may interact with *TGFBR2*, thereby participating in the transformation of NASH to HCC. Given the significant role of *GDF15* in mediating fibrosis, strategies aimed at modulating its expression or activity could provide therapeutic benefits. By inhibiting *GDF15* or blocking its pathways, it might be possible to reduce fibrogenesis and thus slow down the progression of NAFLD to more severe forms. This approach holds promise, particularly in patients who exhibit high levels of *GDF15* and are therefore at increased risk of disease progression.

The integration of bioinformatics with clinical research offers a promising pathway to unravel the complex molecular underpinnings of diseases like NASH and HCC. Future studies should focus on validating these findings in larger, multicentric cohorts to enhance the generalizability and clinical applicability of these potential biomarkers.

## Conclusion

This study contributes to the growing body of evidence supporting the genetic basis of NASH progression to HCC. The identification of key genes provides a foundation for future research into their biological functions and interactions within the liver microenvironment. By continuing to explore these genetic markers, we can advance the development of targeted therapies and improve prognostic assessments, ultimately enhancing patient management and outcomes in NASH and HCC.

## Data Availability

The datasets presented in this study can be found in online repositories. The names of the repository/repositories and accession number(s) can be found in the article/**Supplementary Material**.
