# Supplementary material for: Biomarkers and potential therapeutic targets driving progression of non-alcoholic steatohepatitis to hepatocellular carcinoma predicted through transcriptomic analysis
Source: Front Immunol. 2024 Dec 4;15:1502263. doi: 10.3389/fimmu.2024.1502263 (PMC11652351; doi:10.3389/fimmu.2024.1502263)
Supplement: Supplementary file 1 [file DataSheet1.docx]

Supplementary Material

Biomarkers and potential therapeutic targets driving progression of non-alcoholic steatohepatitis to hepatocellular carcinoma predicted through transcriptomic analysis

Hui Fan^1†^, Rong Wang^2†^, Bin Wen^1^, Jing Xiong^1^*

^1^Department of Pharmacology, School of Pharmacy, China Pharmaceutical University, Nanjing 210009, China

^2^State Key Laboratory of Natural Medicines, School of Traditional Chinese Pharmacy, China Pharmaceutical University, Nanjing 210009, China

*** Correspondence author:**

Jing Xiong, Ph.D., Department of Pharmacology, School of Pharmacy, China Pharmaceutical University, 24 Tongjiaxiang, Nanjing, Jiangsu 210009, China.

E-mail: [jxiong@cpu.edu.cn](mailto:jxiong@cpu.edu.cn)

†These authors have contributed equally to this work


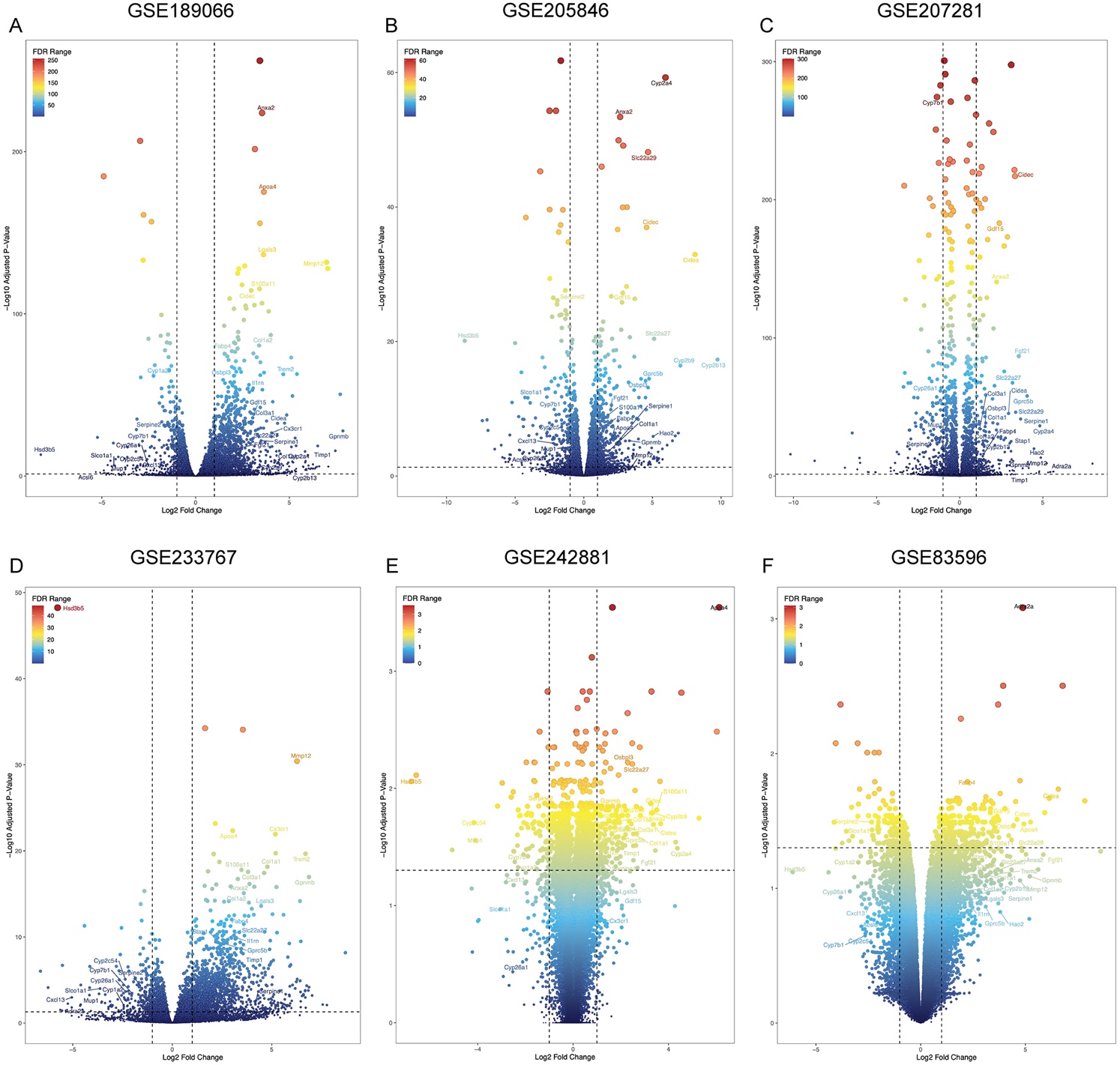


**Supplementary Figure 1.** Volcano plots displaying hub genes in GSE189066 **(A)**, GSE205846 **(B)**, GSE207281 **(C)**, GSE233767 **(D)**, GSE242881**(E)** and GSE83596 **(F)**.


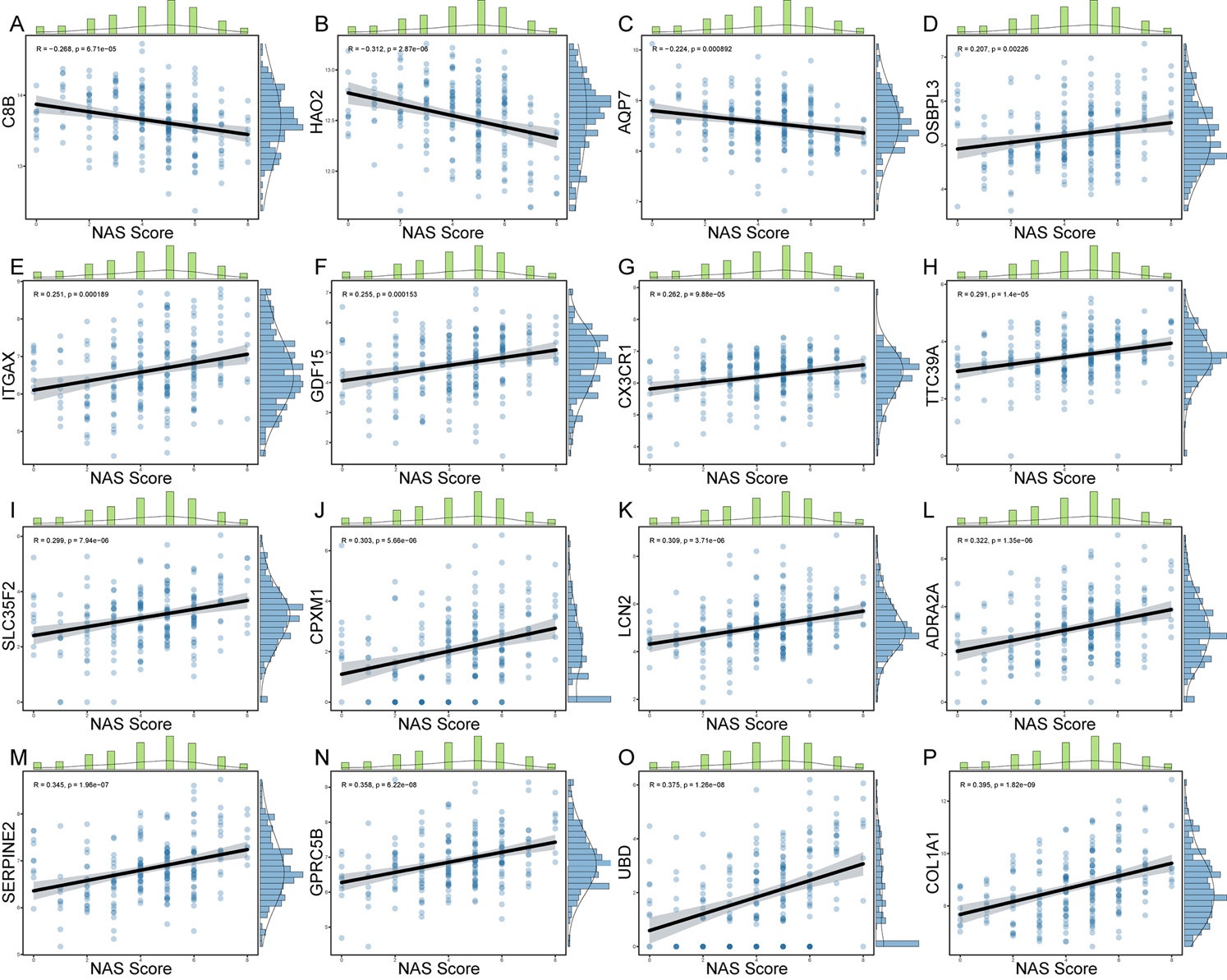


**Supplementary Figure 2.** Correlation analysis of genes identified via RRA with NAS score in GSE135251. Scatter plots for genes selected by RRA that have correlation coefficients between -0.3 and -0.2 **(A–C)**, between 0.2 and 0.3 **(D–I)** and between 0.3 and 0.4 **(J–P)** with NAS score.

**
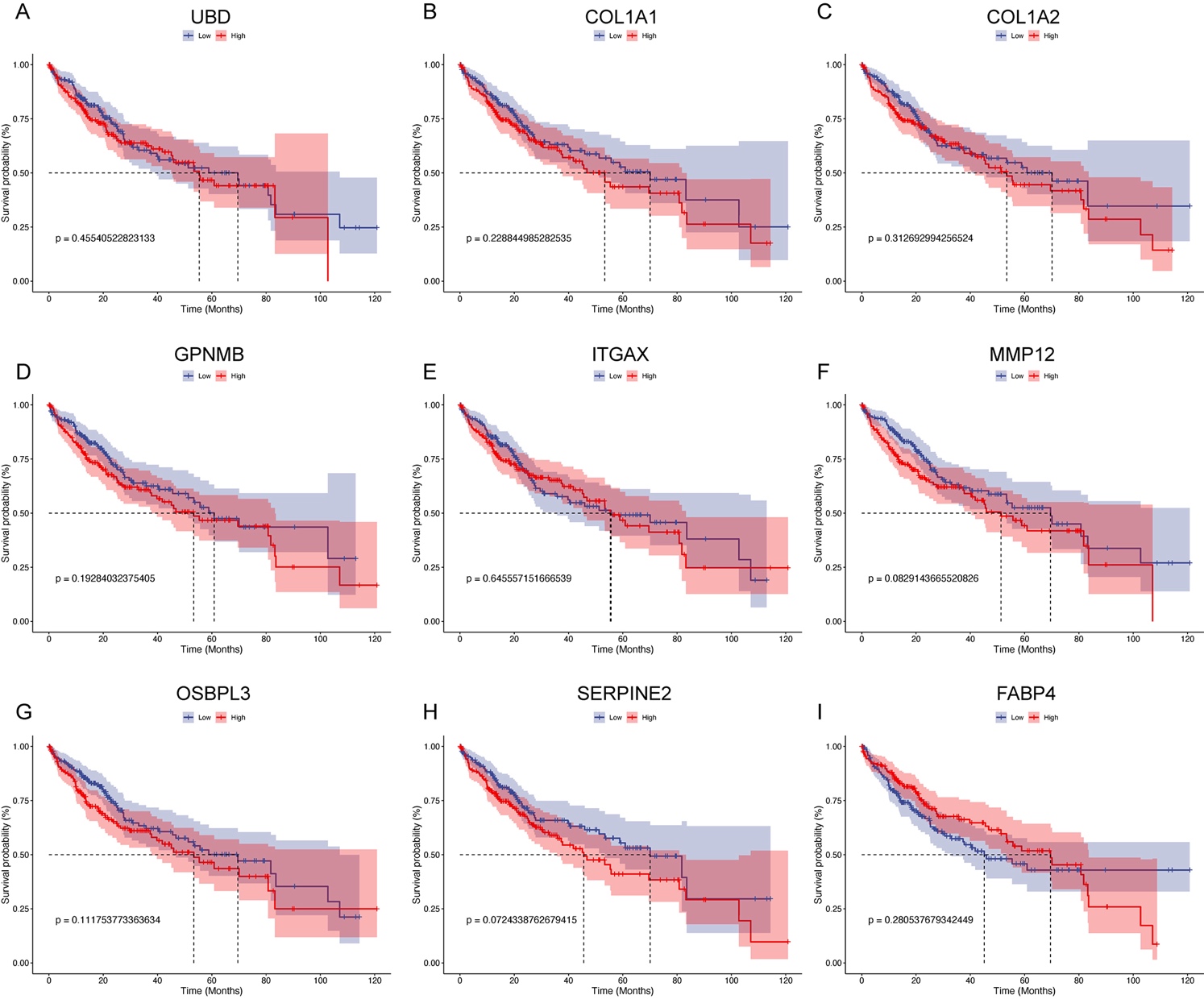
**

**Supplementary Figure 3.** Kaplan-Meier survival curves for *UBD* **(A)**, *COL1A1* **(B)**, *COL1A2* **(C)**, *GPNMB* **(D)**, *ITGAX* **(E)**, *MMP12* **(F)**, *OSBPL3* **(G)**, *SERPINE2* **(H)**,and *FABP4* **(I)** in TCGA liver cancer dataset, categorized into high and low expression groups based on the median expression levels of each gene.


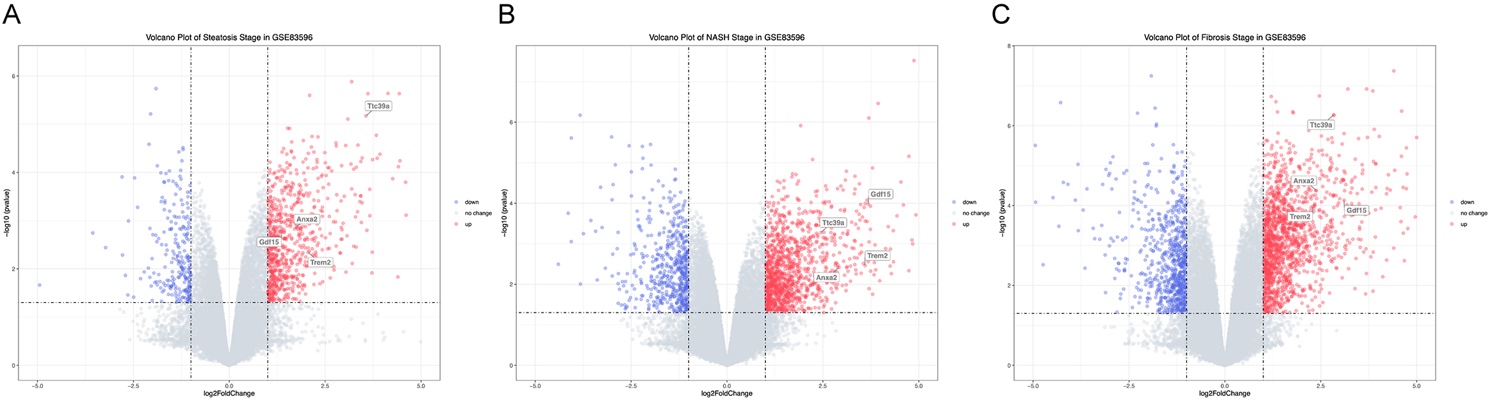


**Supplementary Figure 4.** Volcano plots illustrating the expression of *Trem2*, *Anxa2*, *Ttc39a* and *Gdf15* in steatosis**(A)**, NASH**(B)**, and fibrosis**(C)** in GSE83596.


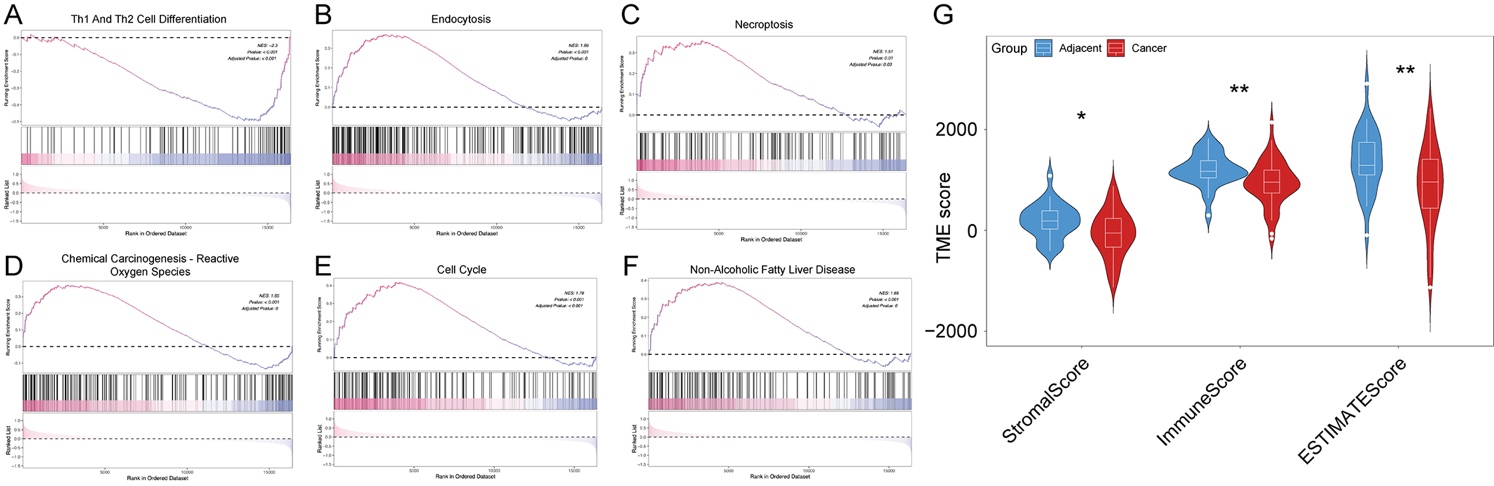


**Supplementary Figure 5.** Gene Set Enrichment Analysis and immune infiltration analysis in NASH-associated hepatocellular carcinoma. **(A–F)** Gene Set Enrichment Analysis (GSEA) for NASH-associated HCC compared with adjacent from dataset GSE164760. **(G)** Violin plots depicting the immune infiltration analysis using the ESTIMATE algorithm for tumor versus adjacent non-tumor tissues in NASH-associated HCC, showing differences in StromalScore, ImmuneScore, and ESTIMATEScore.


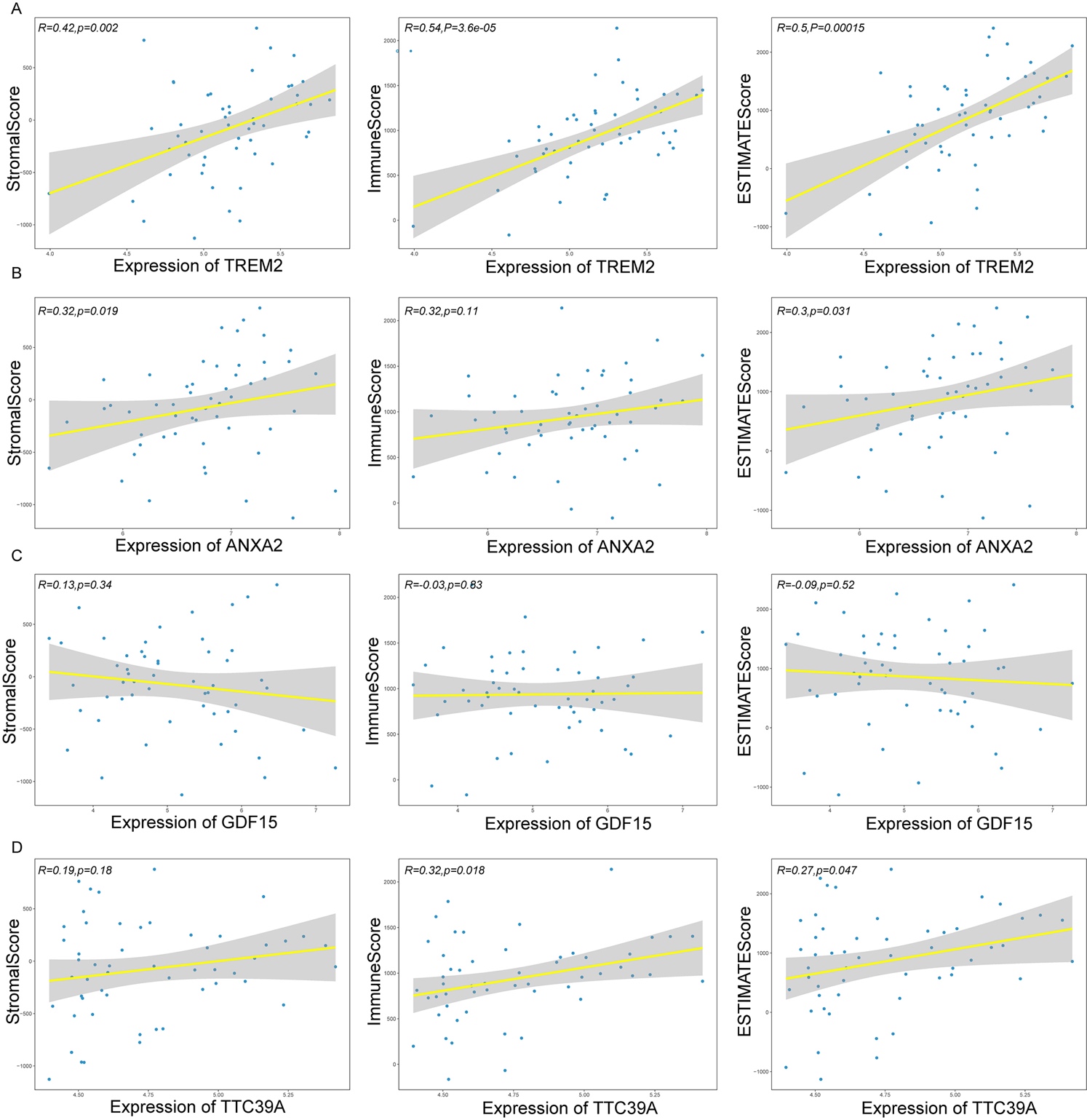


**Supplementary Figure 6.** **(A–D)** Correlation scatter plots illustrating the relationship between the expression levels of *TREM2*, *ANXA2*, *GDF15*, and *TTC39A* and immune-related scores (StromalScore, ImmuneScore, and ESTIMATEScore) across the samples.


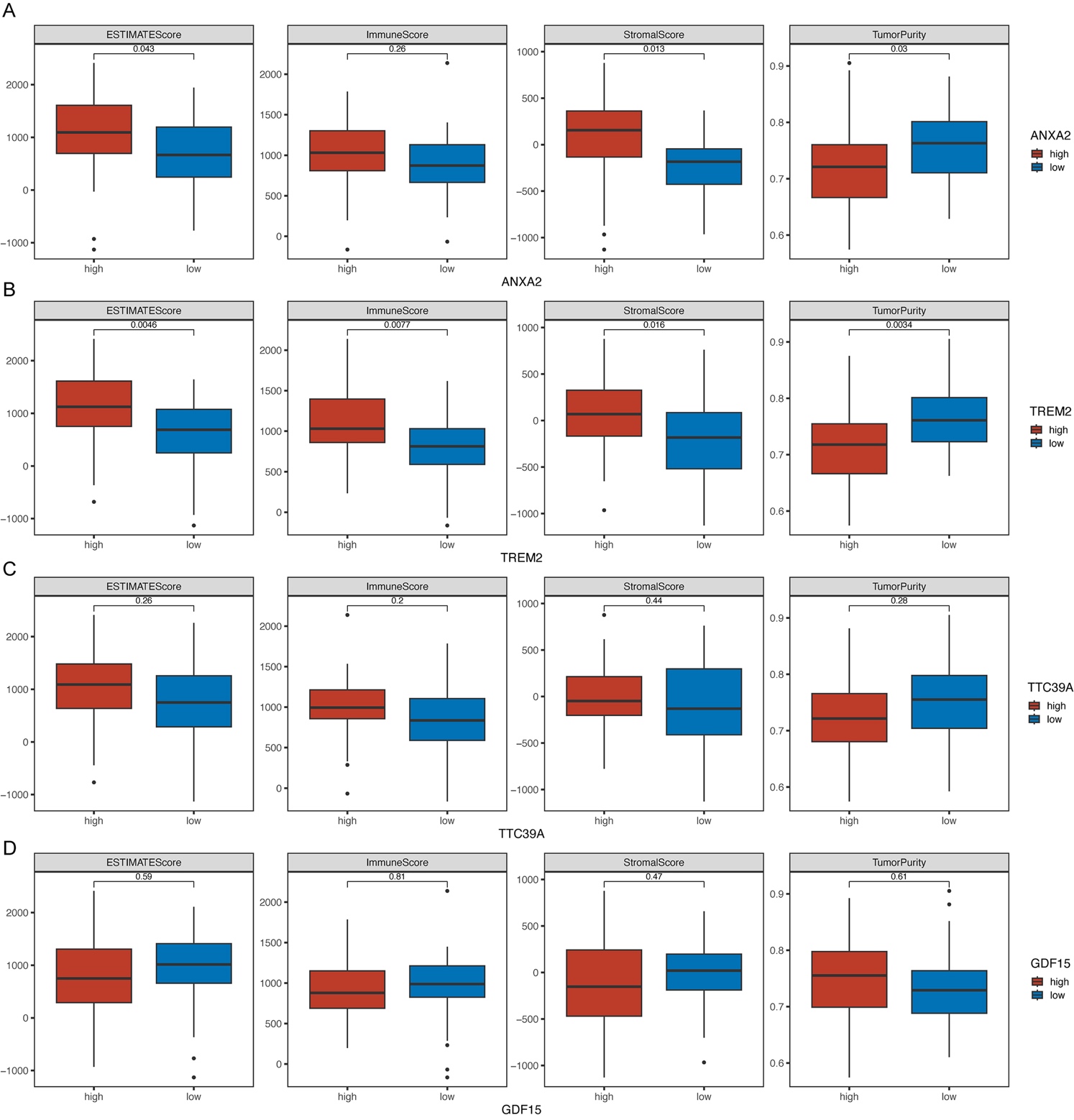


**Supplementary Figure 7.** Differences in immune scores and stromal scores were analyzed in NASH-associated HCC patients with high and low expression of *ANXA2* (A), *TREM2* (B), *TTC39A* (C) and *GDF15* (D).


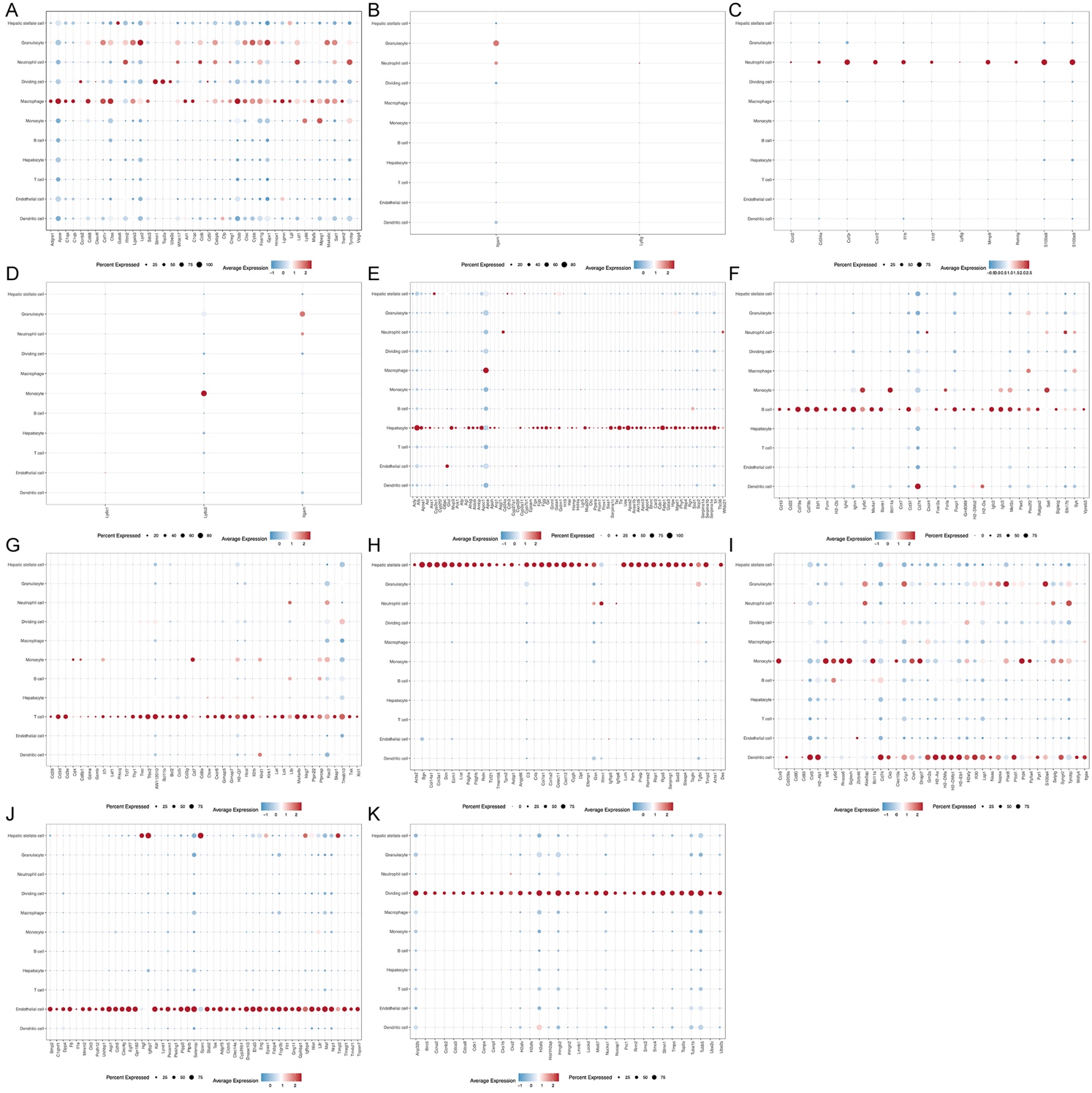


**Supplementary Figure 8.** Dotplot visualization of marker gene expression for Macrophage**(A)**, Granulocyte**(B)**, Neutrophil cell**(C)**, Monocyte**(D)**, Hepatocyte**(E)**, B cell**(F)**, T cell**(G)**, Hepatic stellate cell**(H)**, Dendritic cell**(I)**, Endothelial cell**(J)** and Dividing cell**(K)**.


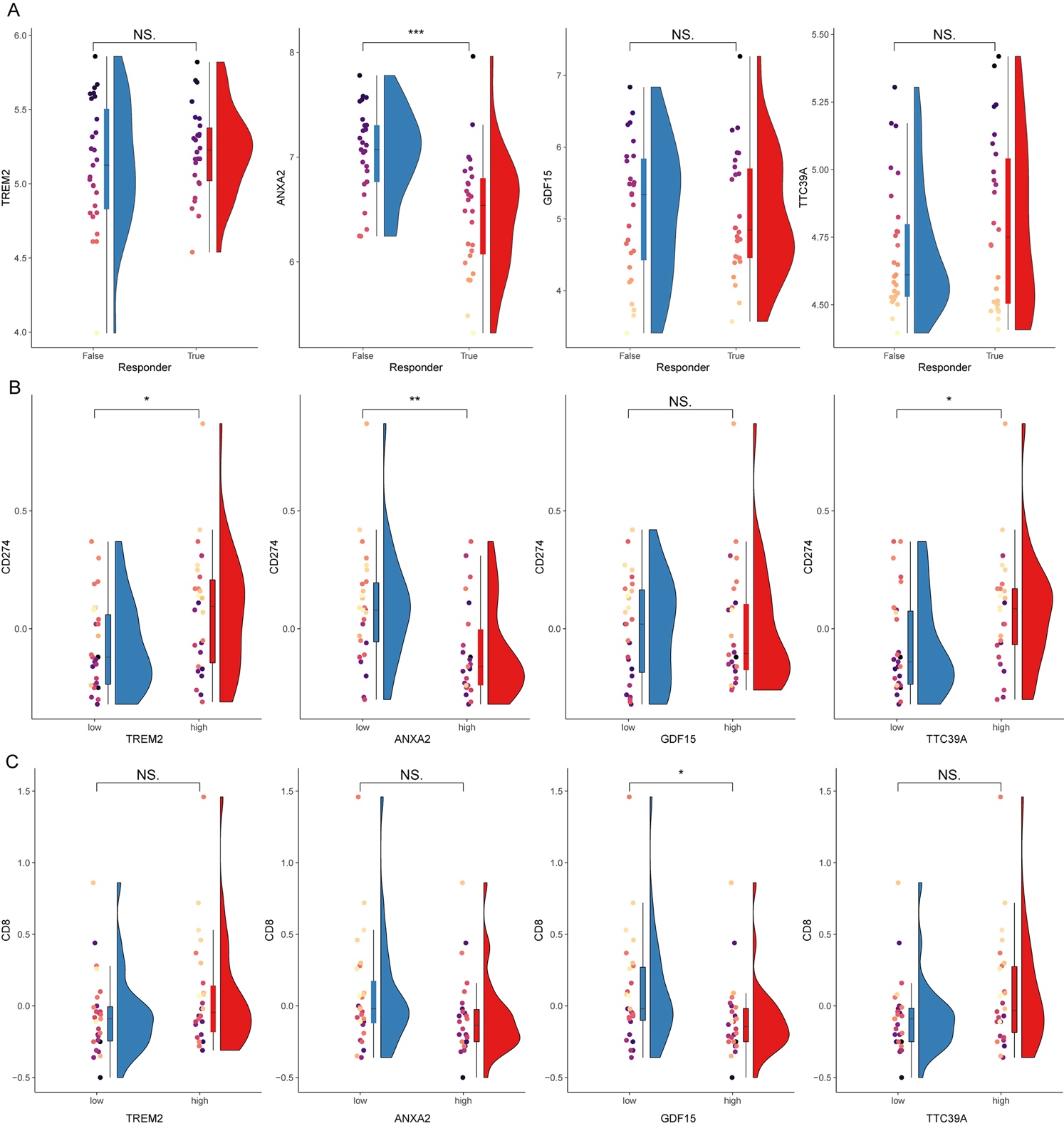


**Supplementary Figure 9.** **(A)** Raincloud plots of *TREM2*, *ANXA2*, *GDF15*, and *TTC39A* expression levels stratified by response to immune therapy. **(B)** Raincloud plots illustrating *CD274* expression levels in high versus low expression groups of the genes. **(C)** Raincloud plots showing *CD8* expression across high and low expression groups of the genes.

**Supplementary TABLE 1 | Forest plot utilizing TCGA liver cancer data to analyze the hazard ratios (HR) of the RRA genes, showing their potential prognostic value in hepatocellular carcinoma.**

| Gene | HR | Lower 95%CI | Upper 95%CI |  | pvalue |
| --- | --- | --- | --- | --- | --- |
| SPRR1A | 1.179 | 0.907 | 1.532 |  | 0.218 |
| LY6D | 0.987 | 0.494 | 1.974 |  | 0.970 |
| SDCBP2 | 1.022 | 0.896 | 1.166 |  | 0.747 |
| MMP12 | 1.190 | 1.037 | 1.367 |  | 0.013 |
| GPNMB | 1.055 | 0.924 | 1.205 |  | 0.430 |
| RBM24 | 1.170 | 0.979 | 1.398 |  | 0.083 |
| AQP7 | 0.964 | 0.722 | 1.286 |  | 0.802 |
| ANXA2 | 1.316 | 1.118 | 1.550 |  | 0.001 |
| CIDEA | 1.004 | 0.787 | 1.281 |  | 0.973 |
| SERPINE1 | 1.125 | 1.022 | 1.239 |  | 0.016 |
| CCDC120 | 1.457 | 1.048 | 2.027 |  | 0.025 |
| MMP13 | 1.262 | 0.717 | 2.222 |  | 0.419 |
| UBD | 1.035 | 0.944 | 1.135 |  | 0.463 |
| COL1A1 | 1.055 | 0.964 | 1.154 |  | 0.247 |
| CIDEC | 1.105 | 0.976 | 1.251 |  | 0.116 |
| RAD51B | 1.712 | 0.795 | 3.690 |  | 0.170 |
| APOA4 | 1.019 | 0.966 | 1.075 |  | 0.492 |
| CDKN1A | 1.006 | 0.869 | 1.165 |  | 0.934 |
| LCN2 | 1.006 | 0.947 | 1.068 |  | 0.849 |
| EPHB2 | 0.997 | 0.802 | 1.239 |  | 0.976 |
| PLS1 | 1.117 | 0.900 | 1.386 |  | 0.315 |
| FAM83A | 0.927 | 0.532 | 1.617 |  | 0.790 |
| PSRC1 | 1.933 | 1.537 | 2.431 |  | 0.000 |
| GPRC5B | 1.174 | 0.986 | 1.397 |  | 0.072 |
| THEMIS | 0.539 | 0.226 | 1.285 |  | 0.163 |
| RGS16 | 1.036 | 0.906 | 1.184 |  | 0.605 |
| S100A11 | 1.193 | 1.069 | 1.332 |  | 0.002 |
| TIMP1 | 1.046 | 0.956 | 1.144 |  | 0.326 |
| GDF15 | 1.089 | 0.974 | 1.217 |  | 0.133 |
| CBR3 | 1.013 | 0.837 | 1.225 |  | 0.898 |
| TTC39A | 1.269 | 1.073 | 1.500 |  | 0.005 |
| COL1A2 | 1.057 | 0.952 | 1.173 |  | 0.297 |
| MAB21L3 | 1.197 | 0.739 | 1.941 |  | 0.465 |
| IL1RN | 0.862 | 0.753 | 0.987 |  | 0.031 |
| COL3A1 | 1.040 | 0.947 | 1.141 |  | 0.415 |
| FGF21 | 0.955 | 0.884 | 1.032 |  | 0.241 |
| HAO2 | 0.932 | 0.862 | 1.007 |  | 0.076 |
| ADRAZA | 0.937 | 0.751 | 1.169 |  | 0.563 |
| EGR2 | 1.031 | 0.782 | 1.361 |  | 0.827 |
| FABP4 | 0.909 | 0.808 | 1.023 |  | 0.115 |
| SLC35F2 | 1.168 | 0.895 | 1.525 |  | 0.253 |
| CX3CR1 | 1.486 | 1.002 | 2.205 |  | 0.049 |
| CPXM1 | 1.193 | 0.970 | 1.467 |  | 0.094 |
| ITGAX | 1.061 | 0.832 | 1.353 |  | 0.632 |
| TREM2 | 1.226 | 1.075 | 1.400 |  | 0.002 |
| TNFRSF10B | 1.069 | 0.840 | 1.362 |  | 0.586 |
| STAP1 | 0.667 | 0.299 | 1.492 |  | 0.324 |
| SLC39A5 | 0.971 | 0.894 | 1.055 |  | 0.491 |
| KRT20 | 1.105 | 0.986 | 1.055 |  | 0.085 |
| CLEC4E | 1.106 | 0.756 | 1.617 |  | 0.604 |
| OSBPL3 | 1.493 | 1.108 | 2.011 |  | 0.008 |
| LGALS3 | 1.182 | 1.056 | 1.324 |  | 0.004 |
| SLC13A2 | 0.952 | 0.795 | 1.140 |  | 0.595 |
| CES4A | 0.872 | 0.709 | 1.072 |  | 0.193 |
| CYP26A1 | 0.859 | 0.648 | 1.139 |  | 0.290 |
| CADM4 | 1.051 | 0.889 | 1.242 |  | 0.561 |
| NAT8 | 1.014 | 0.910 | 1.130 |  | 0.798 |
| ACSL6 | 0.748 | 0.577 | 0.971 |  | 0.029 |
| GRM8 | 1.737 | 1.232 | 2.451 |  | 0.002 |
| SERPINE2 | 1.184 | 1.027 | 1.364 |  | 0.020 |
| CYP7B1 | 0.995 | 0.827 | 1.198 |  | 0.959 |
| CYP1A2 | 0.956 | 0.885 | 1.033 |  | 0.256 |
| C8B | 0.867 | 0.786 | 0.956 |  | 0.256 |
| CXCL13 | 1.047 | 0.937 | 1.169 |  | 0.415 |
|  |  |  |  |   Hazard Ratios |  |
