# Supplementary figures and images for: Biomarkers and potential therapeutic targets driving progression of non-alcoholic steatohepatitis to hepatocellular carcinoma predicted through transcriptomic analysis
Source: Front Immunol. 2024 Dec 4;15:1502263. doi: 10.3389/fimmu.2024.1502263 (PMC11652351; doi:10.3389/fimmu.2024.1502263)

Original Western blot bands in Figure 5M


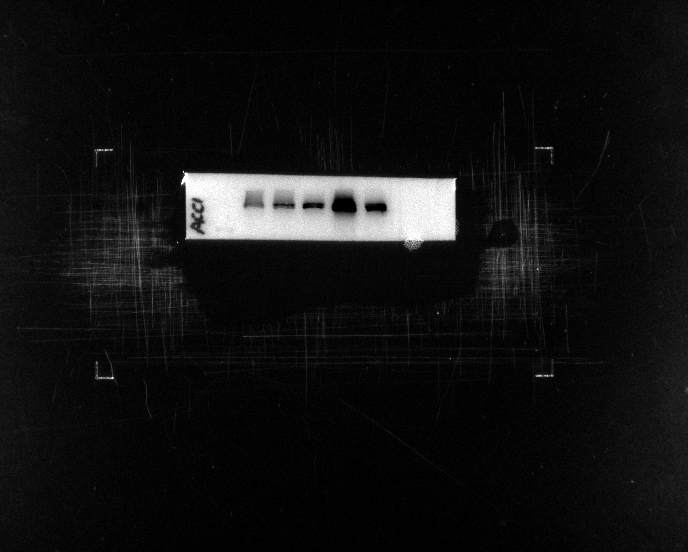

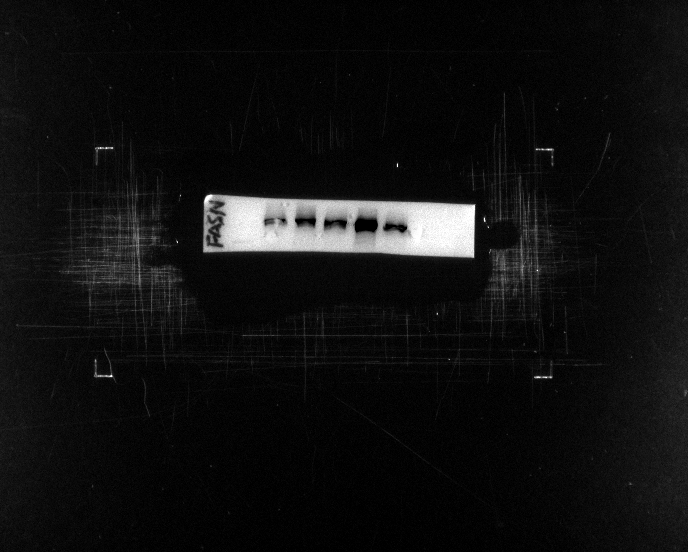


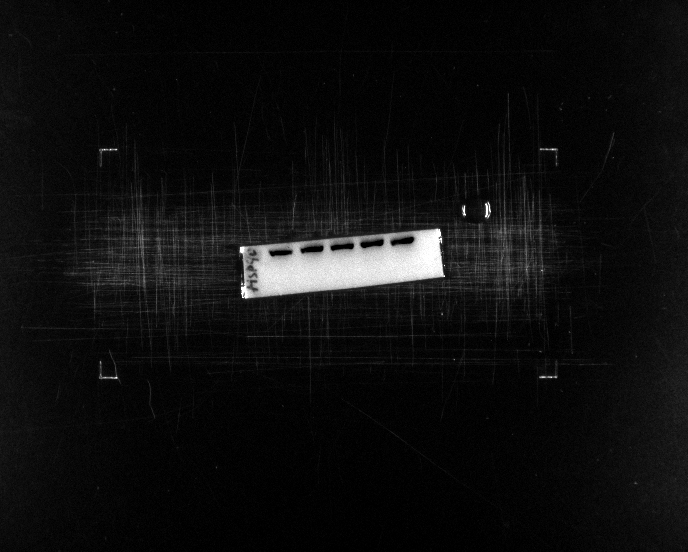

Supplement: Supplementary file 2 [file DataSheet2.docx]
